# Neural Representation of Chinese Word Classes: An ALE Meta-Analysis of Noun and Verb Processing

**DOI:** 10.3390/bs16101886

**Published:** 2026-10-13

**Authors:** Xiaoyang Luo, Yu Deng, Yi Sun

**Affiliations:** 1 Center for Linguistics and Applied Linguistics, Guangdong University of Foreign Studies, Guangzhou 510420, China; 2 Center for Linguistic, Literary & Cultural Studies, Sichuan International Studies University, Chongqing 400031, China; dengyu@sisu.edu.cn

**Keywords:** Chinese word classes, noun–verb processing, ALE meta-analysis, grammatical category, lexical semantics, neuroimaging

## Abstract

Whether nouns and verbs rely on distinct neural systems remains debated, particularly in Chinese, which has limited inflectional morphology and flexible word-class use. We used activation likelihood estimation in GingerALE 3.0.2 to synthesize task-based fMRI/PET studies identified from three databases. Twenty-three publications contributed 28 experiment-level contrast entries. The primary analyses compared 8 noun-versus-baseline and 15 verb-versus-baseline entries, using a pooled 23-entry reference; the original mixed inputs of 9 noun-associated and 19 verb-associated entries were retained as additional analyses. Input maps used cluster-level family-wise error inference at *p* = 0.05, and final between-set contrasts used *p* = 0.05 with a 200 mm^3^ minimum cluster size. Noun-related convergence was concentrated in the left parietal region, whereas verb-related convergence involved frontal and posterior regions. The primary comparison identified greater noun-related convergence in the left parietal and right claustral/insular regions and greater verb-related convergence in bilateral frontal and right posterior regions. The baseline-only conjunction yielded no suprathreshold cluster, while the additional analyses showed a broadly comparable pattern. Together, these findings support partly differentiated neural processing of nouns and verbs in Chinese.

## 1. Introduction

Nouns and verbs are central categories in the study of language and cognition. Nouns commonly denote entities and relatively stable concepts, whereas verbs more often encode actions, events, and relations. Whether these categories rely on partly distinct neural systems has long been debated. Early interest arose from observations of language-impaired patients: some showed selective difficulty in producing or retrieving nouns, whereas others were more impaired with verbs. These patterns suggested that grammatical class might be relevant to the organization of lexical representation and motivated the noun–verb dissociation hypothesis.

Clinical findings alone cannot establish the source of an apparent noun–verb dissociation. The observed differences may reflect grammatical class, object-action semantics, imageability, lexical frequency, or task demands. Pulvermüller et al. (1999b) showed that action-related nouns patterned more closely with action verbs than with visually associated nouns, indicating that semantic associations can shape apparent category effects. Similarly, studies of aphasia and semantic dementia suggested that apparent verb deficits may be reduced or eliminated when frequency and imageability are controlled (Bird et al., 2000, 2003). Thus, patient evidence supports the possibility of noun–verb differentiation while also raising the question of whether the differentiation reflects grammatical categories themselves or correlated semantic variables.

Cognitive–neuroscientific studies have likewise provided evidence for both differentiation and overlap. Electrophysiological research has identified category-related temporal effects, although these effects are sensitive to semantic associations, ambiguity, and task context (Pulvermüller et al., 1999a; Barber et al., 2010). PET and fMRI studies have added spatial evidence by identifying stronger verb-related activation in frontal, premotor, or lateral temporal regions and stronger noun-related activation in temporal or occipitotemporal regions (Shapiro et al., 2005, 2006). Other work has emphasized shared lexical–semantic networks or has suggested that differentiation emerges only when particular grammatical or semantic demands are engaged. Semantic and grammatical variables are often difficult to separate (Vigliocco et al., 2006), and cortical differentiation may depend on whether grammatical functions are explicitly activated in context (Tyler et al., 2008). More recent reviews therefore characterize noun–verb effects as the joint outcome of grammatical class, semantic and pragmatic properties, distributional cues, and task demands (Vigliocco et al., 2011; Kemmerer, 2014).

Chinese offers an informative setting for evaluating these competing accounts. Unlike many Indo-European languages, Chinese has limited inflectional marking of word class and permits flexible syntactic uses. Word-class interpretation consequently depends to a greater extent on lexical meaning, syntactic position, and context. These properties make Chinese a useful test case for examining whether noun and verb processing shows partly differentiated spatial convergence or a more overlapping neural organization. The present study asks where reported activation coordinates converge across Chinese noun- and verb-processing experiments and whether the available evidence permits an inference about their neural relationship. We address three questions: (1) where do noun- and verb-related contrasts each show spatial convergence; (2) where do the assembled noun and verb datasets differ or overlap in convergence; and (3) how do differences in task, baseline, and contrast choice influence the interpretation of these patterns?

## 2. Theoretical Framework

Chinese provides a distinctive theoretical setting for the noun–verb debate. In many Indo-European languages, inflectional morphology can mark grammatical class, whereas Modern Chinese has limited inflectional marking and often relies on lexical meaning, syntactic position, and context to identify a word’s function. Nouns typically profile entities and reference and verbs typically profile processes, predication, and participant relations, but these semantic, syntactic, and cognitive features overlap and vary with task demands (Langacker, 1987; Vigliocco et al., 2011; Kemmerer, 2014). Within Chinese linguistics, separation accounts retain distinct noun and verb categories (Wu, 2023; Lu, 2023; Jin & Wang, 2025). Integration accounts emphasize context-dependent classification (J. X. Li, 1924; Gao, 1948). Inclusion accounts propose a broader nominal category in which verbs may be included under certain analyses (Shen, 2015a, 2015b). Accordingly, Chinese provides an informative linguistic context for examining the noun–verb distinction under conditions of limited morphological marking and flexible syntactic use.

Chinese empirical studies have also produced evidence for both noun–verb dissociation and noun–verb integration. Early evidence came from language pathology: Bates et al. (1991) observed dissociable object- and action-naming patterns in Chinese-speaking aphasic patients, suggesting that noun–verb differences can arise even in a language without rich inflectional morphology; Chen and Bates (1998) further showed that such dissociations may appear in both Broca’s and Wernicke’s aphasia. Subsequent studies extended this question to neurologically healthy speakers using neurocognitive methods, especially ERP/EEG and functional neuroimaging. ERP/EEG evidence for noun–verb separation can be organized into semantic and grammatical accounts. On the semantic side, noun–verb differences have been linked to contrasts between object and action meanings, concreteness, imageability, and the semantic biases of ambiguous words (T. Liu et al., 2011; Xia et al., 2014, 2016, 2022; Zhao et al., 2017; J. Feng et al., 2019; Xia & Peng, 2022). On the grammatical side, other studies have suggested that syntactic context and grammatical-category information can also guide Chinese noun–verb dissociation, indicating that the distinction is not reducible to semantics alone (Y. M. Yang et al., 2002; T. Liu & Yang, 2008; S. W. Feng et al., 2020). However, evidence for noun–verb overlap has also been reported: P. Li et al. (2004) found distributed and largely overlapping neural representations of Chinese nouns and verbs, Tsai et al. (2009) reported similar N400 concreteness effects for Chinese nouns and verbs, and J. Yang et al. (2011b) found little neural differentiation between nouns and verbs in late Chinese–English bilinguals. Neuroimaging studies are therefore important because they add a spatial dimension to this debate by localizing category-related activity within distributed cortical networks. Yet, the imaging evidence remains similarly mixed: some fMRI studies support overlap or integration (P. Li et al., 2004; J. Yang et al., 2011b), whereas others report verb-related activation in left lateral temporal, inferior frontal, prefrontal, superior temporal, or motor-related regions after controlling lexical–semantic variables or manipulating syntactic and semantic processing demands (Yu et al., 2011; Y. Zhang et al., 2018; S. W. Feng et al., 2020). Together, these findings suggest that Chinese nouns and verbs are neither simply identical nor necessarily separated by a fixed anatomical boundary; rather, their neural relationship may depend on the interaction among grammatical category, semantic properties, syntactic context, task demands, and imaging modality. More recently, a Chinese representational similarity analysis identified grammatical-class information in the left posterior middle temporal gyrus during an explicit word-class task, illustrating that univariate activation differences and representational information need not localize to the same regions (W. J. Zhang et al., 2024).

Experimental paradigms can emphasize different components of noun and verb processing. Picture naming couples lexical retrieval to visual and object/action processing; verb generation increases selection and retrieval demands; and lexical decisions, semantic judgments, and sentence tasks vary in contextual and syntactic demands. Accordingly, category-associated convergence pooled across paradigms may reflect both word-class-related processing and the demands used to elicit it. A contrast against rest or a visual baseline is therefore not equivalent to a matched within-study noun–verb contrast. Activation likelihood estimation (ALE) tests whether reported coordinates converge spatially across experiments while accounting for spatial uncertainty (Laird et al., 2005; Eickhoff et al., 2009, 2012, 2016). Earlier cross-language syntheses reported category-associated convergence (H. Yang et al., 2017; Faroqi-Shah et al., 2018), but the Chinese evidence warrants a language-specific assessment that explicitly records these design differences.

We use separate ALE maps for noun-versus-baseline and verb-versus-baseline datasets and compare these baseline-only datasets as the primary between-set analysis, including a conjunction restricted to the same baseline maps. We retain the broader mixed noun-associated and verb-associated datasets as additional analyses. Separation accounts would be compatible with reproducibly different category-associated convergence; integration accounts would be compatible with substantial overlap; and inclusion accounts suggest an asymmetric relation (Shen, 2015a, 2015b). We therefore evaluate the primary and additional patterns together with the number of contributing experiments and their task differences.

## 3. Materials and Methods

### 3.1. Identification and Selection of Studies

The literature search and study selection procedures were conducted and reported in accordance with the PRISMA 2020 guidelines (Page et al., 2021). A systematic literature search was conducted in English- and Chinese-language databases from database inception to June 2026. For the English-language search, Web of Science Core Collection and PubMed were searched using database-specific field tags but the same conceptual structure, which combined terms for Chinese/Mandarin, noun–verb or lexical-category processing, and neuroimaging. The Web of Science search was: TS = ((Chinese OR Mandarin) AND (noun* OR verb* OR “lexical category” OR “grammatical class”) AND (neuroimag* OR fMRI OR “functional magnetic resonance imaging” OR PET OR “brain imaging”)), which identified 178 records. The PubMed search was: (Chinese[Title/Abstract] OR Mandarin[Title/Abstract]) AND (noun*[Title/Abstract] OR verb*[Title/Abstract] OR “lexical category”[Title/Abstract] OR “grammatical class”[Title/Abstract]) AND (neuroimag*[Title/Abstract] OR fMRI[Title/Abstract] OR “functional magnetic resonance”[Title/Abstract] OR PET[Title/Abstract] OR “brain imaging”[Title/Abstract]), which identified 95 records. After duplicate removal across the two English-language databases, 183 unique English-language records were retained.

To identify potentially eligible Chinese-language studies not indexed in the English-language databases, China National Knowledge Infrastructure (CNKI) was also searched from database inception to June 2026. The CNKI search used multiple combinations of three Chinese term blocks: Chinese-language materials, noun–verb or word-class terms, and neuroimaging terms. The main Chinese search strategy was: (汉语 OR 中文 OR 普通话 OR 汉字) AND (名词 OR 动词 OR 名动 OR 词类 OR 语法类别 OR 词汇类别) AND (fMRI OR PET OR 磁共振 OR 功能磁共振 OR 脑成像 OR 正电子发射断层扫描). Additional broad searches combined “fMRI”, “PET”, and “磁共振” with Chinese noun–verb and word-class terms to reduce the risk of missing relevant records. The CNKI search identified 89 records. In total, 272 records were entered into the initial screening stage, including 183 records from the English-language databases and 89 records from CNKI.

The 272 records identified from the database searches were screened by title, abstract, and keywords. Initial screening was conducted using deliberately broad criteria to minimize the risk of excluding potentially eligible neuroimaging studies before full-text assessment. Records were retained for full-text review if they met at least one of the following criteria: (1) the study reported, or appeared likely to report, task-based fMRI or PET evidence involving Chinese or Mandarin language processing; (2) the study involved noun–verb processing, lexical category processing, grammatical class processing, verb generation, noun/object processing, action semantics, object semantics, or related paradigms that could potentially yield noun- or verb-related activation coordinates; (3) the title, abstract, or keywords suggested that healthy Chinese or Mandarin speakers might have been included; or (4) the abstract did not provide enough information to determine whether whole-brain stereotactic coordinates for eligible baseline or direct noun–verb contrasts were available. Records were excluded at this stage if they clearly met any of the following criteria: (1) the study was unrelated to language, lexical, semantic, or grammatical processing; (2) the study did not use task-based fMRI or PET; (3) the article was a review, theoretical paper, commentary, protocol, or other non-original empirical report; (4) the study clearly focused only on clinical, neurological, psychiatric, or otherwise non-healthy samples; (5) the study clearly did not involve Chinese-language materials, Chinese/Mandarin speakers, or Chinese/Mandarin language processing; or (6) the available title and abstract clearly indicated that no noun-, verb-, word-class-, object-, or action-related neural activation data could be extracted. Coordinate availability, whole-brain analysis, and contrast eligibility were not treated as strict exclusion criteria during initial screening because these details were often absent from abstracts and were therefore evaluated during full-text assessment. After this broad initial screening, 113 records remained for full-text assessment, including 94 records from the English-language databases and 19 records from CNKI.

**Figure 1:**
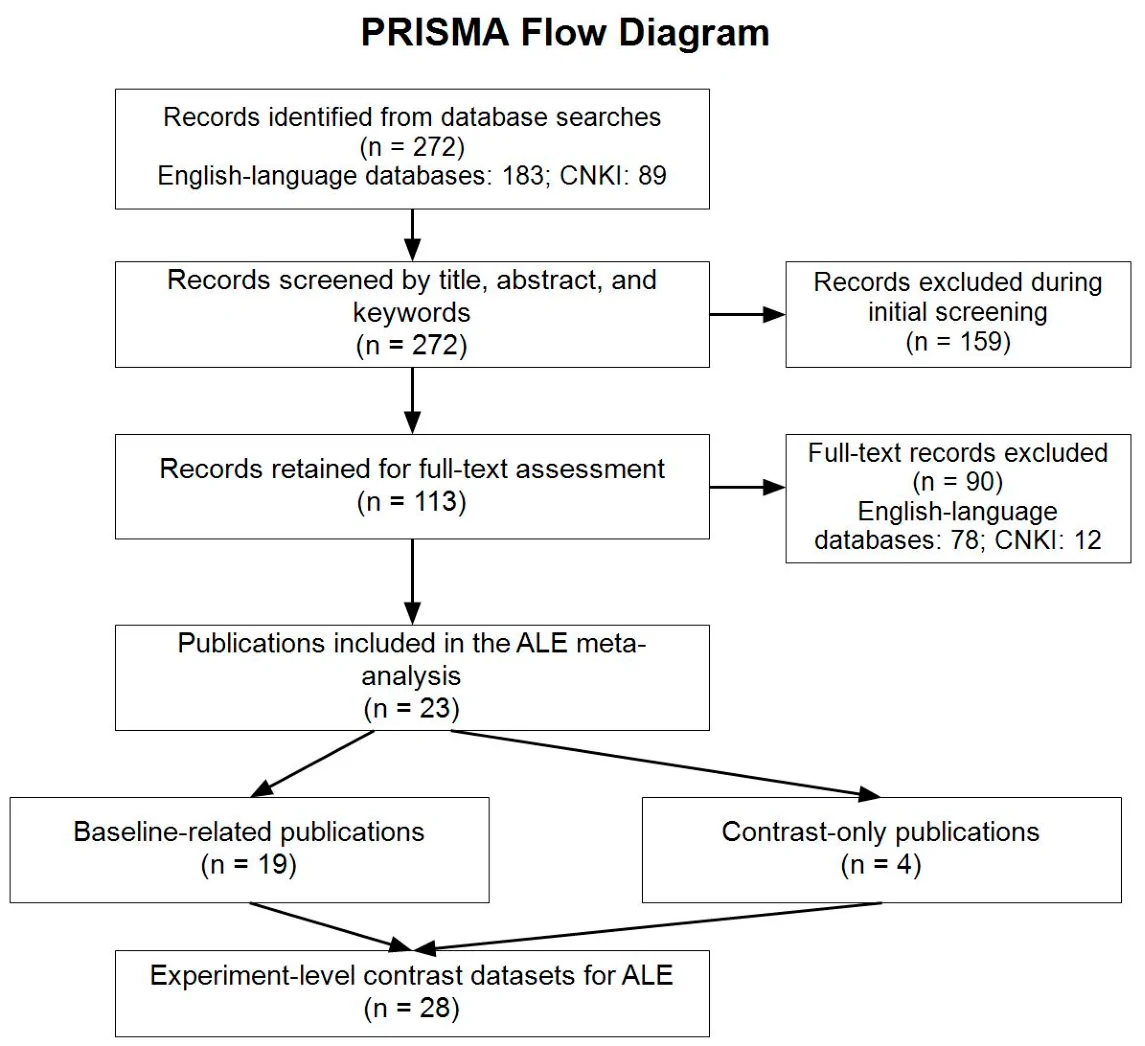
PRISMA flow diagram of the literature search and study selection process.

The 113 records retained after initial screening were subsequently assessed at the full-text level. Full-text eligibility was determined according to the following criteria: (1) the study included healthy Chinese- or Mandarin-speaking participants; (2) the experimental materials were Chinese-language words, phrases, or sentences; (3) the study used task-based fMRI or PET rather than EEG/ERP, behavioral testing, structural imaging, resting-state imaging, or connectivity-only analyses; (4) the study reported original empirical data rather than a review, theoretical discussion, commentary, or methodological protocol; (5) the neuroimaging analysis was based on whole-brain activation rather than exclusively on predefined regions of interest; and (6) the article reported extractable MNI or Talairach stereotactic coordinates for eligible noun- or verb-versus-baseline contrasts or direct noun–verb contrasts that could be coded for ALE analysis. Studies were excluded if they failed to meet any of these criteria. Of the 113 full-text records assessed, 90 were excluded, including 78 records from the English-language databases and 12 records from CNKI. Twenty-three publications met the final eligibility criteria and were included in the ALE meta-analysis, including 16 publications from the English-language databases and 7 publications from CNKI. Among these, 19 publications reported eligible noun- or verb-versus-baseline coordinates, whereas 4 publications reported only direct noun–verb contrast coordinates. The 23 eligible publications yielded 28 experiment-level contrast datasets for ALE analysis. The study selection process is shown in Figure 1, and the eligible publications are summarized in Table 1.

### 3.2. ALE Meta-Analysis Procedures

The revised analysis framework distinguished primary baseline-only analyses from additional mixed-input analyses in GingerALE 3.0.2. The noun-versus-baseline map used 8 experiment-level noun-versus-baseline (N > B) datasets, and the verb-versus-baseline map used 15 verb-versus-baseline (V > B) datasets. The primary between-set comparison contrasted these 8 N > B and 15 V > B datasets. A pooled baseline-only map containing 23 entries, 414 foci, and 355 participant entries served as the reference map for the primary comparison/conjunction workflow, and the same baseline-only maps entered the primary conjunction. As an additional analysis, the noun-associated input A comprised the 8 N > B datasets plus the original noun-greater-than-verb (N > V) coordinates from Yu et al. (2011), giving 9 entries. The verb-associated input B comprised the 15 V > B datasets plus original verb-greater-than-noun (V > N) coordinates from Yu et al. (2011), Y. Zhang et al. (2018), S. W. Feng et al. (2020), and T. Wang and Yang (2017), giving 19 entries. A pooled 28-entry map served as the reference for these additional mixed-input comparisons and their conjunction. Study-to-contrast assignments and analysis specifications are provided in Supplementary File S1.

The analyses followed recommendations for coordinate-based neuroimaging meta-analysis (Müller et al., 2018). ALE models each reported focus as a three-dimensional Gaussian distribution and tests spatial convergence across experiments while accounting for spatial uncertainty (Turkeltaub et al., 2002; Laird et al., 2005; Eickhoff et al., 2009, 2012). Coordinates were analyzed in MNI space; originally Talairach coordinates were converted to MNI space (Lancaster et al., 2007). Analyses used the gray-matter mask specified in the saved GingerALE outputs and 1000 permutations for threshold estimation or between-set comparison, as applicable.

The implemented analysis parameters were as follows. For the N > B and V > B maps and the baseline-only and mixed-input pooled maps used for comparison, cluster-level family-wise error (FWE) inference was set at *p* = 0.05, with an uncorrected cluster-forming threshold of *p* = 0.05 and 1000 permutations. The final between-set contrast maps in both comparison workflows used an uncorrected voxel-level *p* = 0.05 threshold, a minimum cluster volume of 200 mm^3^, and 1000 permutations. The primary conjunction assessed spatial overlap between the baseline-only N > B and V > B ALE maps, with the pooled 23-entry map serving as the pooled reference. The additional conjunction assessed the mixed A and B maps, with the pooled 28-entry map serving as its reference.

## 4. Results

The 23 eligible publications contributed 28 experiment-level contrast entries. The N > B analysis included 132 foci from 8 entries (109 participant entries), and the V > B analysis included 282 foci from 15 entries (246 participant entries). These baseline-only datasets formed the primary between-set analysis; their pooled input contained 414 foci from 23 entries (355 participant entries). The additional noun-associated input A contained 140 foci from 9 entries (130 participant entries), and the additional verb-associated input B contained 316 foci from 19 entries (323 participant entries). Their mixed pooled input contained 456 foci from 28 entries (453 participant entries). Only one included publication contributed N > V coordinates and four contributed V > N coordinates (Table 1; Supplementary File S1).

The N > B and V > B input maps showed different spatial extents under the implemented cluster-level inference threshold of *p* = 0.05. Noun-associated convergence was concentrated in a left parietal cluster, whereas the verb-associated map contained frontal, cerebellar, occipital, and temporal clusters. In the primary baseline-only comparisons, N > B greater than V > B yielded two clusters and V > B greater than N > B yielded three clusters. The baseline-only conjunction yielded no suprathreshold cluster. The additional mixed-input analyses showed broadly comparable dominant anatomical patterns, and their conjunction likewise yielded no suprathreshold cluster. Table 2 lists the saved output coordinates and volumes.

### 4.1. Category-Specific Activation Relative to Baseline

The noun-versus-baseline analysis yielded one suprathreshold cluster with a volume of 11,936 mm^3^ under the implemented cluster-level FWE *p* = 0.05 threshold. This left parietal cluster was centered in the inferior parietal lobule/postcentral region (center: −39, −40.3, 46.6; peak: −40, −40, 46; ALE = 0.0152; Z = 4.56). Anatomical labeling also included the superior parietal lobule, precuneus, and supramarginal gyrus, with the largest contribution from Brodmann area 40.

The verb-versus-baseline analysis yielded four suprathreshold clusters with a combined volume of 57,856 mm^3^ under the same threshold. The largest was in the left frontal lobe (21,152 mm^3^; peak: −52, 4, 46; ALE = 0.0293; Z = 6.11), involving precentral, middle frontal, and inferior frontal gyri. Other clusters involved right cerebellar/occipital areas (15,576 mm^3^), bilateral medial/superior frontal and cingulate areas (11,976 mm^3^), and left cerebellar/ventral temporal areas (9152 mm^3^).

**Figure 2:**
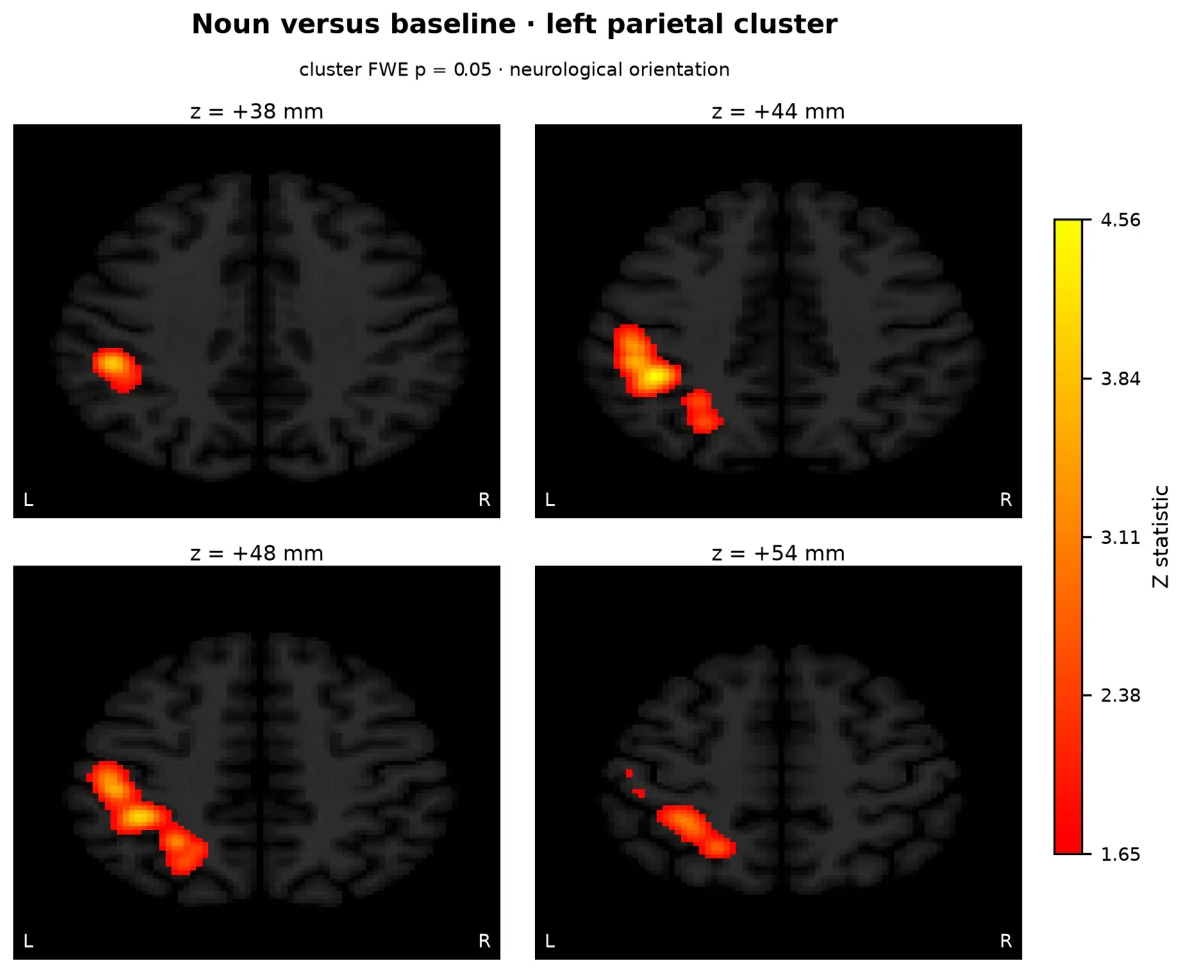
Left parietal cluster from the noun-versus-baseline ALE map on four axial MNI slices (z = +38, +44, +48, and +54 mm). Color shows Z values inside the saved cluster mask.

**Figure 3:**
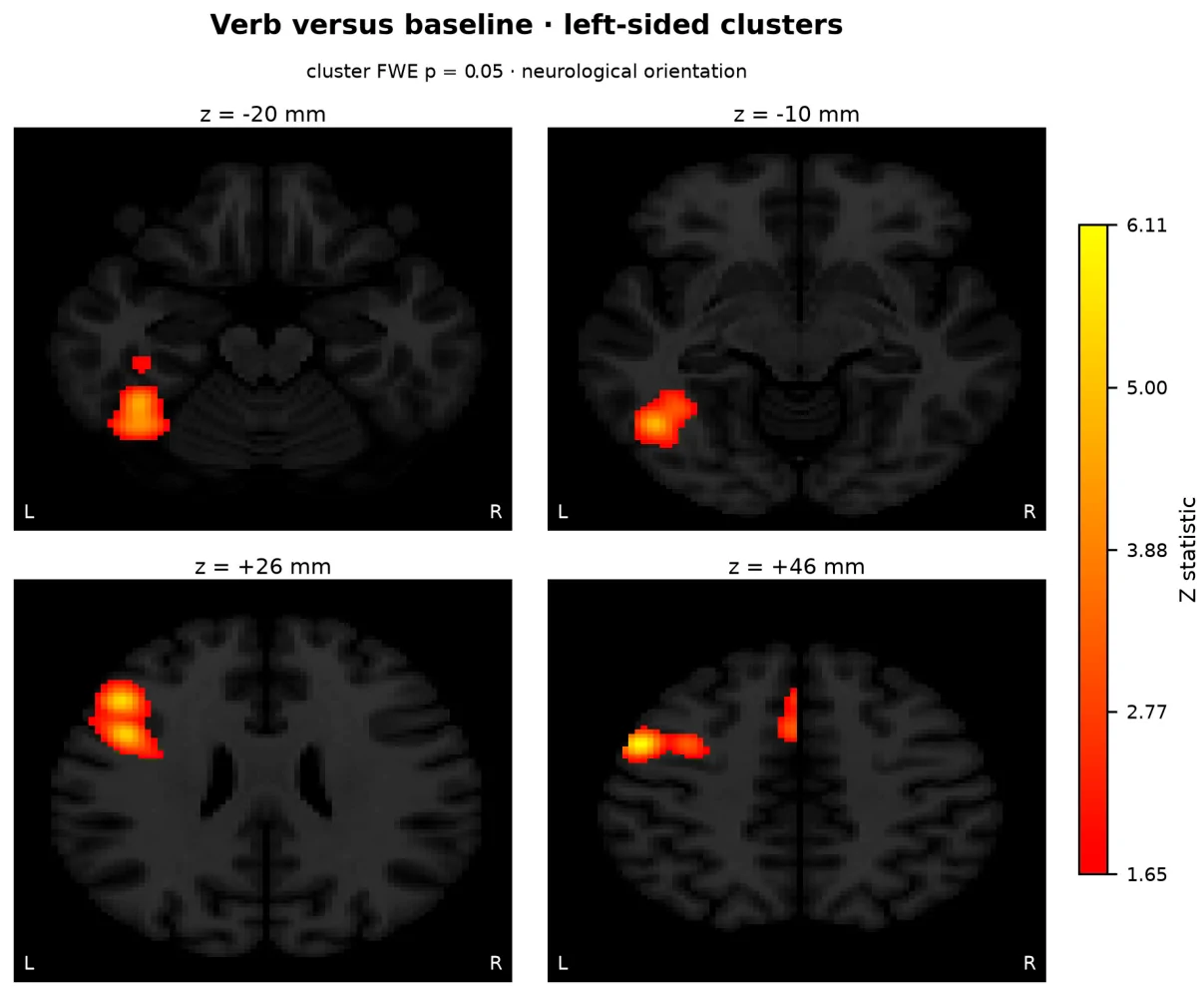
Left-sided clusters from the verb-versus-baseline ALE map on four axial MNI slices (z = −20, −10, +26, and +46 mm). Color shows Z values inside the saved cluster mask.

**Figure 4:**
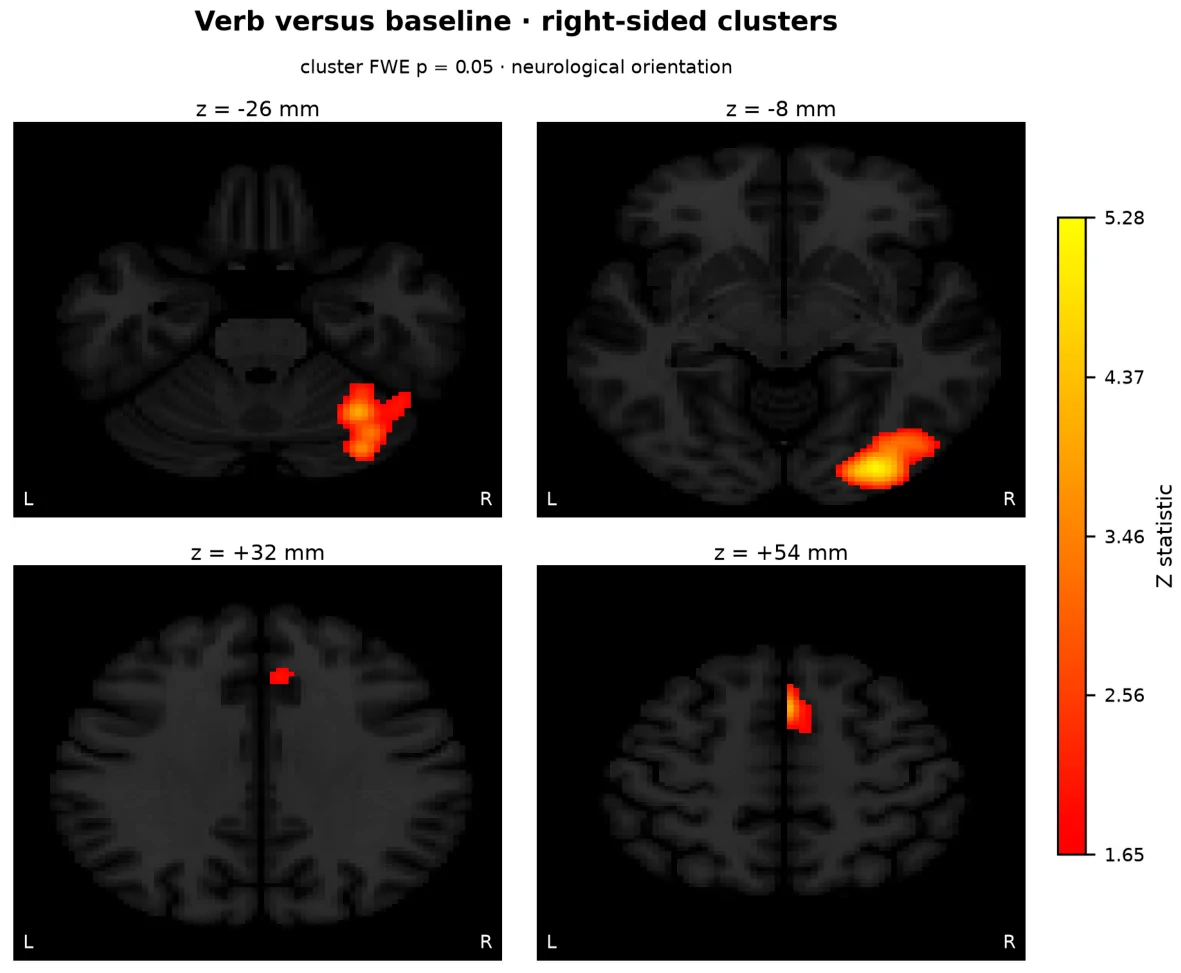
Right-sided clusters from the verb-versus-baseline ALE map on four axial MNI slices (z = −26, −8, +32, and +54 mm). Color shows Z values inside the saved cluster mask.

The baseline-related maps therefore differed in observed spatial extent, although the noun input contained fewer experiments than the verb input (8 versus 15). Figure 2, Figure 3 and Figure 4 provide multi-slice axial views of the baseline-related clusters.

### 4.2. Primary Baseline-Only Comparisons of Noun- and Verb-Related Convergence

The primary baseline-only N > B greater than V > B comparison yielded two clusters with a total volume of 5072 mm^3^ at uncorrected *p* = 0.05 with a 200 mm^3^ minimum cluster size. The larger cluster was located in the left inferior parietal/postcentral region (4784 mm^3^; peak: −46, −36.5, 46.3; Z = 3.29), extending into the supramarginal gyrus. A smaller cluster was identified in the right claustral/insular region (288 mm^3^; peak: 31, 28.3, −0.3; Z = 2.07).

The primary baseline-only V > B greater than N > B comparison yielded three clusters with a total volume of 7272 mm^3^ under the same threshold and minimum size. The largest cluster was located in the left frontal region (3560 mm^3^; peak: −37.7, 6.3, 33.2; Z = 3.29), involving the precentral, inferior frontal, and middle frontal gyri. The other clusters were located in the right occipital/posterior cerebellar region (2192 mm^3^; peak: 36, −78, −14; Z = 3.29) and the right frontal region (1520 mm^3^; peak: 54.9, 5.7, 40.6; Z = 3.09).

**Figure 5:**
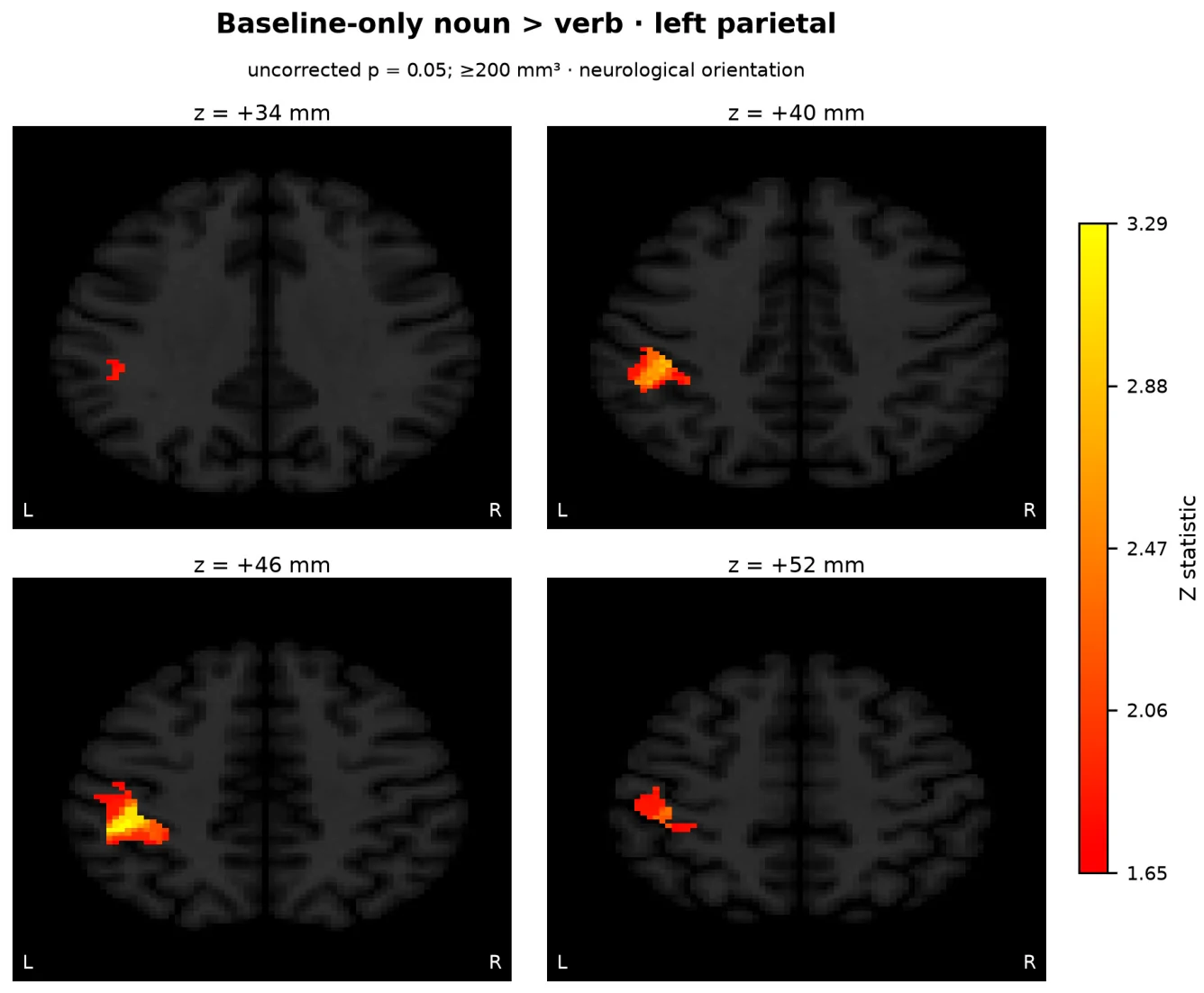
Left parietal cluster in the primary baseline-only N > B > V > B ALE comparison, shown on four axial MNI slices (z = +34, +40, +46, and +52 mm). Color shows Z values inside the saved cluster mask.

**Figure 6:**
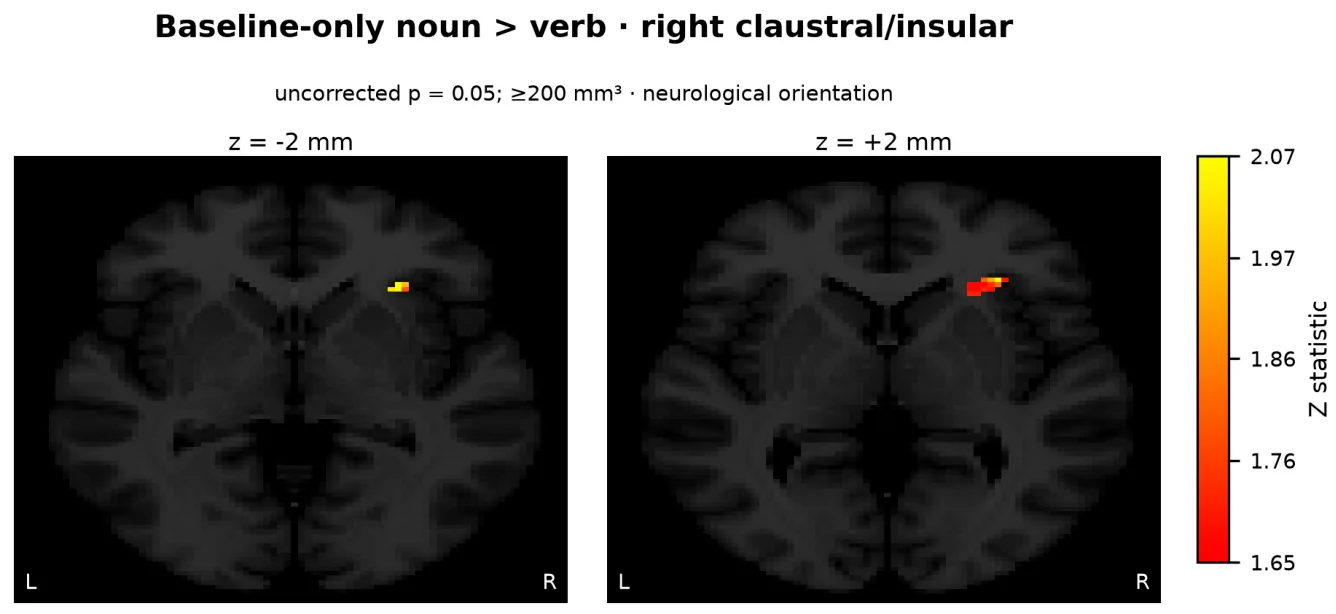
Right claustral/insular cluster in the primary baseline-only N > B > V > B ALE comparison, shown on two axial MNI slices (z = −2 and +2 mm). Color shows Z values inside the saved cluster mask.

**Figure 7:**
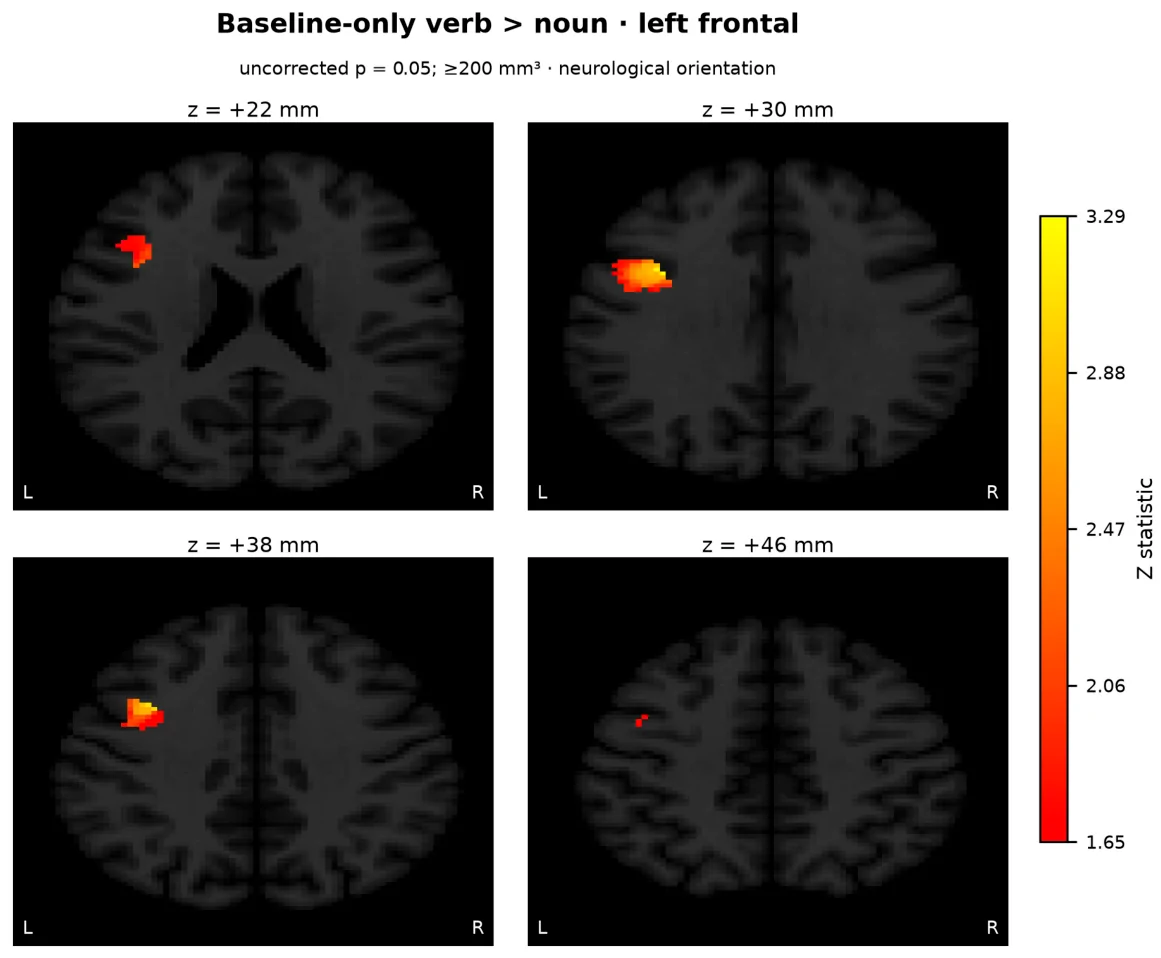
Left frontal cluster in the primary baseline-only V > B > N > B ALE comparison, shown on four axial MNI slices (z = +22, +30, +38, and +46 mm). Color shows Z values inside the saved cluster mask.

**Figure 8:**
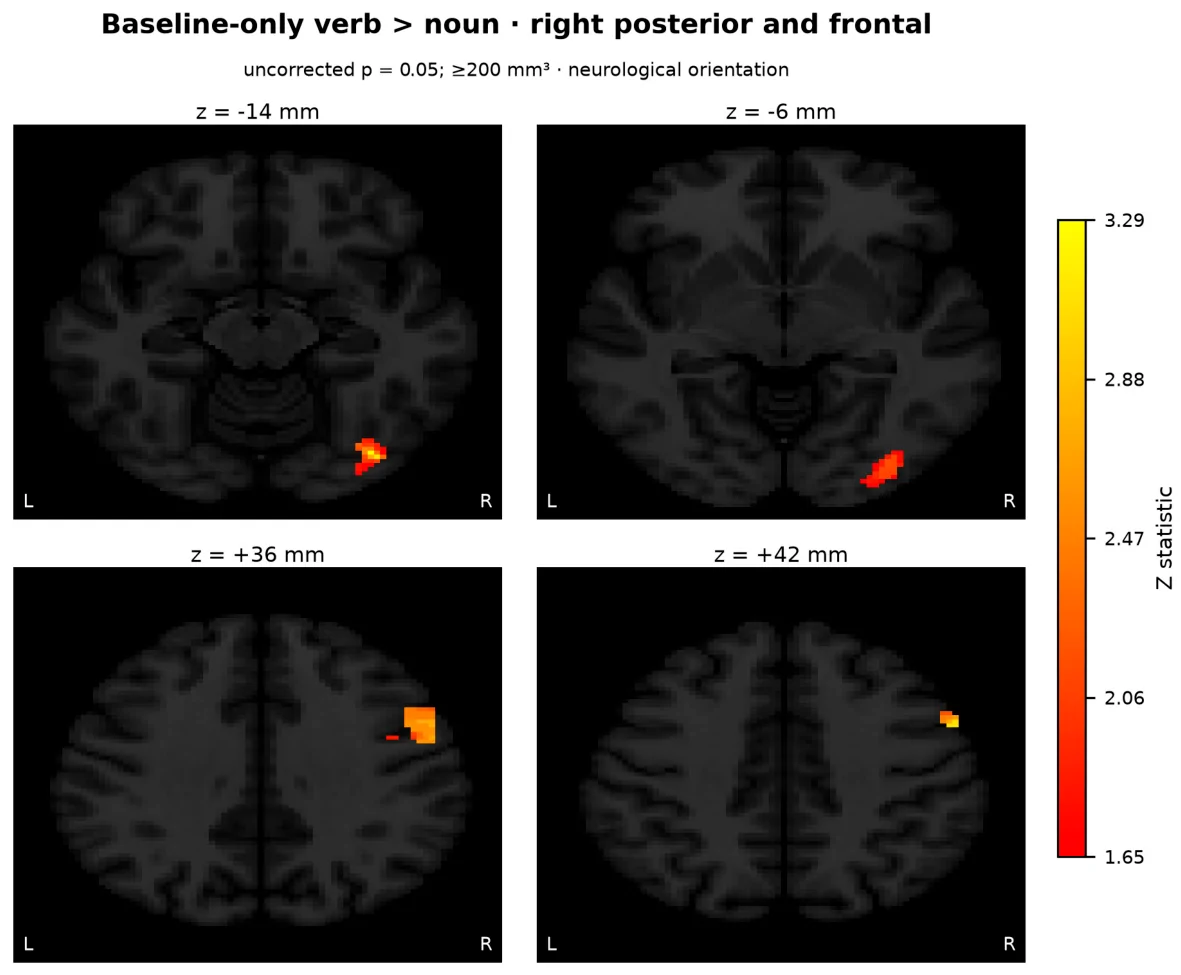
Right occipital/posterior cerebellar and right frontal clusters in the primary baseline-only V > B > N > B ALE comparison, shown on four axial MNI slices (z = −14, −6, +36, and +42 mm). Color shows Z values inside the saved cluster mask.

The two primary baseline-only between-set maps displayed different dominant anatomical patterns. Figure 5, Figure 6, Figure 7 and Figure 8 present multi-slice axial views of these maps.

### 4.3. Primary Baseline-Only Conjunction

The conjunction of the baseline-only N > B and V > B ALE maps yielded no suprathreshold cluster under the implemented analysis settings.

### 4.4. Additional Mixed-Input Analyses

The additional mixed-input analyses retained the original noun-associated input A (8 N > B entries plus 1 N > V entry; 9 entries) and verb-associated input B (15 V > B entries plus 4 V > N entries; 19 entries). The A > B comparison yielded five clusters totaling 5624 mm^3^, including a dominant left inferior parietal/postcentral cluster. The B > A comparison yielded five clusters totaling 7104 mm^3^, with dominant left and right frontal and right posterior patterns. These maps also contained several smaller clusters that were not retained in the primary baseline-only comparisons. The conjunction of the mixed-input maps yielded no suprathreshold cluster. Detailed coordinates are provided in Supplementary File S1.

## 5. Discussion

This ALE meta-analysis identified different spatial convergence patterns in the assembled Chinese noun- and verb-associated datasets. The primary baseline-only comparisons identified greater noun-related convergence predominantly in the left parietal region, with a smaller right claustral/insular cluster, and greater verb-related convergence in bilateral frontal and right posterior regions. The additional mixed-input analyses showed a broadly comparable anatomical organization. These findings provide preliminary evidence of partial differentiation in noun- and verb-associated neural processing. Interpretation of this pattern must nevertheless take into account the unequal inputs and the heterogeneity of tasks and baselines.

### 5.1. Neural Mechanisms of Chinese Noun and Verb Processing

The noun-related ALE analysis revealed a relatively focal convergence pattern, mainly involving the left parietal region. This finding suggests that Chinese noun processing may depend on neural mechanisms associated with lexical–semantic integration and the mapping between written word forms and conceptual representations. Neuroimaging evidence has shown that the inferior parietal cortex, including the angular and supramarginal regions, forms part of a heteromodal semantic network that supports conceptual integration across modality-specific information (Binder et al., 2009; Binder & Desai, 2011). In the present study, the left parietal convergence for nouns may therefore reflect the integration of entity-based semantic features rather than a purely grammatical representation of nouns.

This interpretation is particularly relevant for Chinese. Because Chinese lacks rich inflectional morphology, noun status is not typically marked by overt morphological cues but is instead determined through lexical meaning, syntactic position, and contextual constraints. Previous Chinese neuroimaging and ERP studies have shown that noun–verb differences can emerge even under minimal morphological marking, indicating that semantic properties play an important role in category-specific processing in Chinese (P. Li et al., 2004; Yu et al., 2011; J. Feng et al., 2019; Xia et al., 2016). In particular, object-relatedness, concreteness, and imageability have been shown to contribute to noun–verb distinctions in Chinese word processing (Xia & Peng, 2022). Thus, the noun-related parietal cluster observed in the present meta-analysis may reflect the processing of object- or entity-based conceptual information together with lexical–semantic integration demands.

The verb-associated map covered more regions than the noun-associated map, a pattern compatible with the action, event, and relational demands often associated with verb tasks. However, the verb map also drew on 15 rather than 8 baseline-related experiment entries, which may increase sensitivity and apparent coverage. Included paradigms ranged from picture naming and verb generation to lexical decisions and semantic judgments, with differing visual, selection, retrieval, and syntactic demands (Crepaldi et al., 2011; Vigliocco et al., 2011; Kemmerer, 2014). The observed extent therefore supports a category-associated difference, but it cannot be assigned to grammatical class alone.

The involvement of frontal and premotor-related regions in verb processing is consistent with evidence linking action and event knowledge to frontal and motor-related systems (Martin et al., 1995; Kable et al., 2002). Chinese fMRI evidence has also shown that action verbs can recruit motor-related neural resources during picture naming, supporting the view that motor features constitute an important dimension of action verb representation (Y. Zhang et al., 2018). However, verb-related frontal activation should not be reduced to motor simulation alone. Action verb concepts are not merely perceptual or motor representations, and frontal regions may also support semantic selection, controlled retrieval, and the integration of event or argument information (Bedny et al., 2008; Binder & Desai, 2011). Thus, the frontal convergence for Chinese verbs may reflect the combined contribution of action-related semantics, semantic control, and event-structural processing.

The temporal and posterior activations observed for verbs may further indicate the contribution of lexical–semantic storage, event representation, and visual–action associations. Temporal regions are central components of the semantic system and have been implicated in the representation of conceptual and event knowledge (Binder et al., 2009; Binder & Desai, 2011; Kable et al., 2002). The occipital and posterior regions, together with cerebellar activation, should be interpreted cautiously, as they may partly reflect the visual, action-related, or task-specific demands of the studies included in the meta-analysis. Previous work has shown that verb-related effects can vary with task demands, semantic reference, and the degree to which verbs are associated with concrete action features (Saccuman et al., 2006; Bedny et al., 2008; Vigliocco et al., 2011). The present verb-related pattern therefore suggests a distributed processing network, but not a simple one-to-one mapping between verbs and motor cortex.

Taken together, the baseline-related analyses provide preliminary evidence that the sampled noun and verb experiments converged in different ways, consistent with partly differentiated processing. The observed pattern motivates matched experimental comparisons that can clarify how category, semantics, and paradigm jointly shape the relative focality of noun and verb representations.

### 5.2. Noun–Verb Dissociation and Its Possible Sources

The primary baseline-only comparison identified greater noun-related convergence in the left parietal region and a smaller right claustral/insular region, whereas greater verb-related convergence was found in bilateral frontal and right posterior regions. The additional mixed-input maps preserved these dominant patterns, although they also contained several smaller clusters not retained in the baseline-only analysis. The persistence of the principal parietal, frontal, and posterior patterns after the direct N > V and V > N entries were removed indicates that the main spatial differentiation was not dependent on those entries. These results support a partly differentiated account of noun- and verb-associated processing in Chinese.

This differentiation should not be interpreted as evidence for a purely grammatical or fully modular separation between nouns and verbs. Previous neurocognitive studies have shown that noun–verb differences are often shaped by several correlated factors, including grammatical class, semantic content, imageability, concreteness, action-relatedness, morphological marking, contextual information, and task demands (Bedny & Thompson-Schill, 2006; Crepaldi et al., 2011; Vigliocco et al., 2011; Kemmerer, 2014). Nouns are typically associated with objects or entities, whereas verbs are more frequently associated with actions, events, temporal relations, and argument structures. Because these semantic and grammatical properties are naturally correlated, neural differences between nouns and verbs may reflect the interaction of multiple processing dimensions rather than grammatical class alone.

The noun-greater-than-verb effect in the left inferior parietal region may be related to entity-based semantic integration and the mapping of lexical forms onto conceptual representations. Previous Chinese studies have suggested that noun–verb differences can emerge even in the absence of rich morphological marking, and that semantic variables contribute substantially to such differences (P. Li et al., 2004; Yu et al., 2011; J. Feng et al., 2019; Xia et al., 2016). The stronger parietal convergence for nouns may therefore reflect the processing of object-related or entity-related conceptual information, especially when lexical meaning must be integrated with task-specific demands. This interpretation is also consistent with evidence that imageability and grammatical class can produce neuroanatomically separable effects during word comprehension, suggesting that semantic properties should be distinguished from grammatical-category effects when interpreting noun–verb differences (Bedny & Thompson-Schill, 2006).

By contrast, the verb-greater-than-noun pattern appears to reflect the greater processing demands associated with action and event representations. Verb processing often requires access to relational information, argument structure, temporal organization, and action/event knowledge. This interpretation is consistent with evidence that action verbs can recruit frontal and motor-related systems, while also engaging broader semantic and control networks (Bedny et al., 2008; Saccuman et al., 2006; Y. Zhang et al., 2018). At the same time, verb-related frontal activation should not be reduced to motor semantics alone. Some studies have reported neural sensitivity to abstract grammatical distinctions between nouns and verbs, including effects in Broca’s area (Sahin et al., 2006), whereas other studies have shown that noun–verb dissociation can depend on morphological markers, task requirements, or contextual constraints (Federmeier et al., 2000; Longe et al., 2007; Tyler et al., 2008; Berlingeri et al., 2008; Siri et al., 2008). Thus, the frontal and posterior convergence observed for verbs in the present study may reflect the combined contribution of action/event semantics, semantic selection, argument-structure processing, and task-dependent control.

Experimental paradigms do more than introduce heterogeneity. They place noun- and verb-associated processing within different linguistic and cognitive operations and can therefore reveal different aspects of their neural differentiation. In picture naming, the same visual stimulus can be named as an object or as an action, making object/action conceptual content and action-related motor features central to the noun–verb contrast (Y. Zhang et al., 2018). Semantic-relatedness judgments instead emphasize access to lexical–semantic relations and have identified verb-associated responses in the left lateral temporal and inferior frontal regions even when relevant lexical variables are controlled (Yu et al., 2011). Phrase-judgment paradigms further embed nouns and verbs in combinatorial contexts, allowing the distinction to be examined under syntactic and semantic conditions; S. W. Feng et al. (2020) reported greater verb-related activation in frontal and superior temporal regions under both conditions. Thus, the varying parietal and frontal/posterior patterns across the present dataset are compatible with partial noun–verb neural differentiation, while indicating that its anatomical expression depends substantially on the semantic content, syntactic frame, and processing demands emphasized by a given design.

### 5.3. Implications, Limitations, and Future Directions

The present findings have important implications for theories of Chinese word classes. In Chinese linguistics, the status of nouns and verbs has long been debated. Some accounts question the validity of the noun–verb dissociation, arguing that Chinese words may not be categorically specified as nouns or verbs outside syntactic context, echoing the traditional principle that word-class membership should be determined by syntactic use rather than by fixed lexical form (J. X. Li, 1924). Other accounts, especially the noun–verb inclusion hypothesis, propose that the Chinese noun category may have broader distributional and conceptual scope, such that verbs can be understood as being included within or closely related to nominal categories under certain grammatical conditions (Shen, 2015a, 2015b; Sun, 2022; Yue, 2022). These theoretical positions are motivated by well-known typological properties of Chinese, including the lack of rich inflectional morphology, flexible syntactic distribution, frequent category ambiguity or conversion, and strong dependence of category interpretation on context.

The present results add neuroimaging evidence relevant to the view that Chinese noun- and verb-associated processing can be differentiated, despite limited overt inflectional marking. They do not decide whether that differentiation is driven by grammatical category, correlated semantic and syntactic properties, or task demands. Thus, separation, integration, and inclusion accounts remain open to tests that better match these factors.

These findings also indicate that future research should examine Chinese word-class processing as a language-specific system rather than directly importing assumptions from Indo-European languages. Because category information in Chinese is distributed across multiple cues, future studies should more carefully separate lexical categories from correlated semantic, syntactic, and task-related factors. In particular, object-relatedness, action-relatedness, concreteness, imageability, argument structure, and contextual constraint should be systematically controlled or manipulated. Other Chinese-specific variables, such as word length, syllabic structure, and category ambiguity, may also influence noun–verb processing and should be considered in experimental design, but these factors require direct empirical testing rather than being inferred from coordinate-based meta-analysis alone.

Several limitations should inform the interpretation and extension of these findings. The baseline-related noun analysis contained 8 experiment entries, compared with 15 for verbs. Consequently, the narrower noun map and the null conjunction may partly reflect lower sensitivity in the noun dataset. The included studies also differed in their tasks, baselines, stimulus materials, and linguistic contexts. This variation is informative because it shows how partial noun–verb differentiation may be expressed under different processing conditions, but it also means that the precise distribution of parietal, frontal, and posterior convergence should be interpreted in relation to the paradigm used in each study. The present ALE synthesis nevertheless provides evidence that these diverse experimental settings converge on partly differentiated noun- and verb-associated processing in Chinese. Future research can test the stability and task specificity of this pattern through matched within-study noun–verb contrasts and sufficiently large paradigm-specific datasets.

## 6. Conclusions

The available Chinese noun- and verb-associated neuroimaging datasets showed different patterns of spatial convergence in the ALE analyses. The noun-versus-baseline map contained a focal left parietal cluster, whereas the verb-versus-baseline map contained frontal and posterior clusters. The primary baseline-only comparison identified greater noun-related convergence in the left parietal region and a smaller right claustral/insular region, whereas greater verb-related convergence was identified in bilateral frontal and right posterior regions. The baseline-only conjunction did not identify a suprathreshold cluster, and the additional mixed-input analyses showed a broadly comparable spatial organization.

The primary baseline-only results and the broadly comparable additional analyses provide preliminary evidence for partly differentiated noun- and verb-associated neural processing in Chinese. The observed differentiation is compatible with category-related differences in processing, while its anatomical expression is likely shaped by the semantic content, syntactic context, and processing demands emphasized by individual experimental paradigms. It therefore supports a limited, non-categorical form of noun–verb differentiation rather than wholly separate neural modules.

The unequal numbers of noun- and verb-related experiments and the heterogeneity of tasks and baselines should be considered when interpreting the relative extent of the observed maps. Even so, the convergence across the assembled studies provides a foundation for further testing how noun–verb differentiation is expressed across semantic, syntactic, and task contexts in Chinese.

## Figures and Tables

**Table 1:** Eligible publications and contrast-level contributions to the activation likelihood estimation analyses.

Study and Technique	Participants	Tasks	Experimental Baseline/Control	NvsB	VvsB	NvsV	VvsN
Klein et al. (1999), PET (H_2_^15^O-water tracer)	7	Chinese-English verb generation	Word repetition		✓		
Luke et al. (2002), fMRI	7	Syntactic and semantic plausibility judgment	Font-size judgment		✓		
Shan et al. (2003), fMRI	9	Chinese semantic processing	Fixation/no-stimulus control	✓			
P. Li et al. (2004), fMRI	8	Lexical decision for Chinese nouns and verbs	Fixation	✓	✓		
Liang et al. (2006), fMRI	6	Visual processing of Chinese noun, verb, and adjective modifiers	Fixation (crosshair)	✓	✓		
S. W. Feng et al. (2007), fMRI	8	Visual processing of Chinese noun, verb, and adjective phrases	Fixation/+ control task	✓			
Chan et al. (2008), fMRI	11	Noun and verb processing in early bilinguals	Fixation	✓	✓		
J. Liu et al. (2009), fMRI	19	Chinese date noun and general noun processing	Rest/fixation +	✓			
J. Yang et al. (2011a), fMRI	20	Tool-use and hand-action verb processing	Rest		✓		
Yu et al. (2011), fMRI	21	Semantic processing of Chinese nouns and verbs	Fixation			✓	✓
Su et al. (2013), fMRI	13	Semantic comprehension of Chinese action idioms	Symbol baseline conditions		✓		
J. Yang and Shu (2014), fMRI	20	Passive reading and motor imagery of hand and tool-use actions	Rest		✓		
Xi et al. (2016), fMRI	15	Verb generation	Initial rest and resting periods		✓		
T. Wang and Yang (2017), fMRI	18	Noun and verb processing in late high-proficiency Chinese-English bilinguals	Fixation + between task blocks				✓
Xi et al. (2017), fMRI	28	Verb generation	Initial rest and resting periods		✓		
Y. Zhang et al. (2018), fMRI	21	Picture naming of action verbs and nouns	Fixation/ISI implicit baseline				✓
S. W. Feng et al. (2020), fMRI	17	Syntactic and semantic processing of nouns and verbs in phrases	Arrow congruency judgment				✓
X. Wang et al. (2021), fMRI	16	Syntactic processing of Mandarin Chinese intransitive verbs	Arrow direction judgment control task		✓		
X. Wang et al. (2023), fMRI	25	Argument-structure processing of Chinese intransitive verbs	Arrow direction judgment control task		✓		
Yan et al. (2024), fMRI	28	Chinese concrete and abstract word memory processing	Mini-block interval/rest	✓			
W. J. Zhang et al. (2024), fMRI	20	Noun–verb distinction in left posterior middle temporal gyrus	Passive viewing of an asterisk	✓	✓		
X. Wang et al. (2026a), fMRI	25	Processing of Chinese transitive and intransitive verbs	Arrow direction judgment control task		✓		
X. Wang et al. (2026b), fMRI	25	Argument mapping of Chinese unaccusative verbs	Arrow direction judgment control task		✓		

Note. NvsB = noun versus baseline; VvsB = verb versus baseline; NvsV = noun versus verb; VvsN = verb versus noun. Rows identify eligible publications, and check marks identify the contrast types they contributed. The 23 publications yielded 28 experiment-level contrast entries; a publication can contribute more than one entry. The five original category contrasts comprise one NvsV entry and four VvsN entries. Full input assignments are listed in Supplementary File S1.

**Table 2:** Results of the baseline maps and primary baseline-only between-set analyses.

Contrast	Cluster	Vol. (mm^3^)	Gyrus, Brodmann Area (BA)	x	y	z	Peak Value	Total Vol. (mm^3^)
Nouns vs. baseline	1	11,936	Left inferior parietal lobule; postcentral gyrus; superior parietal lobule; precuneus; supramarginal gyrus (BA40; BA7; BA2; BA3)	−40	−40	46	ALE = 0.0152; Z = 4.56	11,936
Verbs vs. baseline	1	21,152	Left precentral gyrus; middle frontal gyrus; inferior frontal gyrus (BA6; BA9; BA46; BA4; BA13; BA45; BA8)	−52	4	46	ALE = 0.0293; Z = 6.11	57,856
	2	15,576	Right cerebellum/occipital region: declive; culmen; fusiform gyrus; lingual gyrus; inferior/middle occipital gyri (BA18; BA19; BA37)	30	−86	−6	ALE = 0.0236; Z = 5.28	
	3	11,976	Bilateral superior frontal gyrus; medial frontal gyrus; cingulate gyrus (BA6; BA32; BA8; BA24)	−2	14	54	ALE = 0.0284; Z = 5.97	
	4	9152	Left cerebellum/ventral temporal region: culmen; fusiform gyrus; declive; parahippocampal gyrus; inferior temporal gyrus (BA37; BA36; BA20; BA19)	−46	−60	−12	ALE = 0.0252; Z = 5.51	
Primary baseline-only N > B > V > B	1	4784	Left inferior parietal lobule; postcentral and supramarginal gyri (BA40; BA2; BA3)	−46	−36.5	46.3	Z = 3.29	5072
	2	288	Right claustrum/insula (BA13)	31	28.3	−0.3	Z = 2.07	
Primary baseline-only V > B > N > B	1	3560	Left precentral gyrus; inferior frontal gyrus; middle frontal gyrus (BA6; BA9; BA46)	−37.7	6.3	33.2	Z = 3.29	7272
	2	2192	Right occipital/posterior cerebellar region: fusiform and occipital gyri; declive (BA18; BA19)	36	−78	−14	Z = 3.29	
	3	1520	Right precentral gyrus; middle frontal gyrus; inferior frontal gyrus (BA6; BA9)	54.9	5.7	40.6	Z = 3.09	
Primary baseline-only conjunction	--	--	No suprathreshold clusters	--	--	--	--	--

Note. Coordinates are in MNI space. Nouns versus baseline and verbs versus baseline used cluster-level FWE inference at *p* = 0.05 after an uncorrected cluster-forming *p* = 0.05 threshold. The primary between-set analyses compared the baseline-only N > B input (8 entries) and V > B input (15 entries), with a pooled 23-entry reference map. The primary N > B > V > B and V > B > N > B contrasts used uncorrected *p* = 0.05 and a minimum cluster volume of 200 mm^3^; the primary conjunction used the same baseline-only maps. Additional mixed-input analyses used A = noun-associated input (9 entries) and B = verb-associated input (19 entries), with a pooled 28-entry reference map. The table reports peak ALE and Z values for the baseline maps and peak Z values for the primary between-set maps. ALE = activation likelihood estimation; FWE = family-wise error. The additional mixed-input results are summarized in Section 4.4 and reported in Supplementary File S1.

## Data Availability

The extracted coordinate inputs, study-to-contrast assignment table, and analysis specifications supporting this study are provided in Supplementary File S1.

## Supplementary Materials

The following supporting information can be downloaded at: https://www.mdpi.com/article/10.3390/bs16101886/s1, Supplementary File S1 contains Tables S1–S3 and Data S1–S5, which provide the study-to-contrast assignments, coordinate inputs, and analysis specifications for the ALE meta-analysis. The included studies are Klein et al. (1999), Luke et al. (2002), Shan et al. (2003), P. Li et al. (2004), Liang et al. (2006), S. W. Feng et al. (2007), Chan et al. (2008), J. Liu et al. (2009), J. Yang et al. (2011a), Yu et al. (2011), Su et al. (2013), J. Yang and Shu (2014), Xi et al. (2016, 2017), T. Wang and Yang (2017), Y. Zhang et al. (2018), S. W. Feng et al. (2020), X. Wang et al. (2021, 2023, 2026a, 2026b), Yan et al. (2024), and W. J. Zhang et al. (2024).

## References

[B1-behavsci-16-01886] Barber H. A., Kousta S.-T., Otten L. J., Vigliocco G. (2010). Event-related potentials to event-related words: Grammatical class and semantic attributes in the representation of knowledge. Brain Research.

[B2-behavsci-16-01886] Bates E., Chen S., Tzeng O., Li P., Opie M. (1991). The noun-verb problem in Chinese aphasia. Brain and Language.

[B3-behavsci-16-01886] Bedny M., Caramazza A., Grossman E., Pascual-Leone A., Saxe R. (2008). Concepts are more than percepts: The case of action verbs. The Journal of Neuroscience.

[B4-behavsci-16-01886] Bedny M., Thompson-Schill S. L. (2006). Neuroanatomically separable effects of imageability and grammatical class during single-word comprehension. Brain and Language.

[B5-behavsci-16-01886] Berlingeri M., Crepaldi D., Roberti R., Scialfa G., Luzzatti C., Paulesu E. (2008). Nouns and verbs in the brain: Grammatical class and task specific effects as revealed by fMRI. Cognitive Neuropsychology.

[B6-behavsci-16-01886] Binder J. R., Desai R. H. (2011). The neurobiology of semantic memory. Trends in Cognitive Sciences.

[B7-behavsci-16-01886] Binder J. R., Desai R. H., Graves W. W., Conant L. L. (2009). Where is the semantic system? A critical review and meta-analysis of 120 functional neuroimaging studies. Cerebral Cortex.

[B8-behavsci-16-01886] Bird H., Howard D., Franklin S. (2000). Why is a verb like an inanimate object? Grammatical category and semantic category deficits. Brain and Language.

[B9-behavsci-16-01886] Bird H., Lambon Ralph M. A., Patterson K., Hodges J. R. (2003). Verbs and nouns: The importance of being imageable. Journal of Neurolinguistics.

[B10-behavsci-16-01886] Chan A. H. D., Luke K. K., Li P., Yip V., Li G., Weekes B., Tan L. H. (2008). Neural correlates of nouns and verbs in early bilinguals. Annals of the New York Academy of Sciences.

[B11-behavsci-16-01886] Chen S., Bates E. (1998). The dissociation between nouns and verbs in Broca’s and Wernicke’s aphasia: Findings from Chinese. Aphasiology.

[B12-behavsci-16-01886] Crepaldi D., Berlingeri M., Paulesu E., Luzzatti C. (2011). A place for nouns and a place for verbs? A critical review of neurocognitive data on grammatical-class effects. Brain and Language.

[B13-behavsci-16-01886] Eickhoff S. B., Bzdok D., Laird A. R., Kurth F., Fox P. T. (2012). Activation likelihood estimation meta-analysis revisited. NeuroImage.

[B14-behavsci-16-01886] Eickhoff S. B., Laird A. R., Grefkes C., Wang L. E., Zilles K., Fox P. T. (2009). Coordinate-based activation likelihood estimation meta-analysis of neuroimaging data: A random-effects approach based on empirical estimates of spatial uncertainty. Human Brain Mapping.

[B15-behavsci-16-01886] Eickhoff S. B., Nichols T. E., Laird A. R., Hoffstaedter F., Amunts K., Fox P. T., Bzdok D., Eickhoff C. R. (2016). Behavior, sensitivity, and power of activation likelihood estimation characterized by massive empirical simulation. NeuroImage.

[B16-behavsci-16-01886] Faroqi-Shah Y., Sampson M., Pranger M., Baughman S. (2018). Neural representation of word categories is distinct in the temporal lobe: An activation likelihood analysis. Human Brain Mapping.

[B17-behavsci-16-01886] Federmeier K. D., Segal J. B., Lombrozo T., Kutas M. (2000). Brain responses to nouns, verbs and class-ambiguous words in context. Brain.

[B18-behavsci-16-01886] Feng J., Gong T., Shuai L., Wu Y. (2019). No morphological markers, no problem: ERP study reveals semantic contribution to distinct neural substrates between noun and verb processing in online sentence comprehension. Frontiers in Neuroscience.

[B19-behavsci-16-01886] Feng S. W., Qi R. N., Yang J., Yu A. Y., Yang Y. M. (2020). Neural correlates for nouns and verbs in phrases during syntactic and semantic processing: An fMRI study. Journal of Neurolinguistics.

[B20-behavsci-16-01886] Feng S. W., Weng X. C., Shen X. A., Lin C. Y., Xiang H. D., Yang Y. M. (2007). Cerebral responses to the visually presented processing of nouns, verbs and adjectives in mandarin Chinese: A functional MRI evaluation. Journal of Clinical Rehabilitative Tissue Engineering Research.

[B21-behavsci-16-01886] Gao M. K. (1948). Hanyu yufa lun *[On Chinese grammar]*.

[B22-behavsci-16-01886] Jin L. X., Wang Z. X. (2025). Some theoretical issues concerning Chinese nouns and verbs: On the noun-verb inclusion theory. Journal of Foreign Languages.

[B23-behavsci-16-01886] Kable J. W., Lease-Spellmeyer J., Chatterjee A. (2002). Neural substrates of action event knowledge. Journal of Cognitive Neuroscience.

[B24-behavsci-16-01886] Kemmerer D. (2014). Word classes in the brain: Implications of linguistic typology for cognitive neuroscience. Cortex.

[B25-behavsci-16-01886] Klein D., Milner B., Zatorre R. J., Zhao V., Nikelski J. (1999). Cerebral organization in bilinguals: A PET study of Chinese-English verb generation. NeuroReport.

[B26-behavsci-16-01886] Laird A. R., Fox P. M., Price C. J., Glahn D. C., Uecker A. M., Lancaster J. L., Turkeltaub P. E., Kochunov P., Fox P. T. (2005). ALE meta-analysis: Controlling the false discovery rate and performing statistical contrasts. Human Brain Mapping.

[B27-behavsci-16-01886] Lancaster J. L., Tordesillas-Gutierrez D., Martinez M., Salinas F., Evans A., Zilles K., Mazziotta J. C., Fox P. T. (2007). Bias between MNI and Talairach coordinates analyzed using the ICBM-152 brain template. Human Brain Mapping.

[B28-behavsci-16-01886] Langacker R. W. (1987). Nouns and verbs. Language.

[B29-behavsci-16-01886] Li J. X. (1924). Xinzhu guoyu wenfa *[New Chinese grammar]*.

[B30-behavsci-16-01886] Li P., Jin Z., Tan L. H. (2004). Neural representations of nouns and verbs in Chinese: An fMRI study. NeuroImage.

[B31-behavsci-16-01886] Liang D. D., Yang Y. M., Feng S. W., Li J. C. (2006). An fMRI study of Chinese noun by nouns, verbs, and adjectives in Chinese. Applied Linguistics.

[B32-behavsci-16-01886] Liu J., Ye Z. X., Jin H., Ling X. Y., Huang L., Mo L. (2009). Brain regions for number involved in the processing of vocabulary of months in Chinese. Acta Physiologica Sinica.

[B33-behavsci-16-01886] Liu T., Ma P. J., Yu L., Liu J. F., Yang Y. M. (2011). An event-related potential study of the noun-verb ambiguous effect in Chinese. Journal of Psychological Science.

[B34-behavsci-16-01886] Liu T., Yang Y. M. (2008). Neural distinction between Chinese nouns and verbs in the grammatical context: An ERP study. Acta Psychologica Sinica.

[B35-behavsci-16-01886] Longe O., Randall B., Stamatakis E. A., Tyler L. K. (2007). Grammatical categories in the brain: The role of morphological structure. Cerebral Cortex.

[B36-behavsci-16-01886] Lu J. M. (2023). More comments on the hypothesis of the “Noun-Verb Inclusion”. Jilin University Journal Social Science Edition.

[B37-behavsci-16-01886] Luke K. K., Liu H. L., Wai Y. Y., Wan Y. L., Tan L. H. (2002). Functional anatomy of syntactic and semantic processing in language comprehension. Human Brain Mapping.

[B38-behavsci-16-01886] Martin A., Haxby J. V., Lalonde F. M., Wiggs C. L., Ungerleider L. G. (1995). Discrete cortical regions associated with knowledge of color and knowledge of action. Science.

[B39-behavsci-16-01886] Müller V. I., Cieslik E. C., Laird A. R., Fox P. T., Radua J., Mataix-Cols D., Tench C. R., Yarkoni T., Nichols T. E., Turkeltaub P. E., Wager T. D., Eickhoff S. B. (2018). Ten simple rules for neuroimaging meta-analysis. Neuroscience & Biobehavioral Reviews.

[B40-behavsci-16-01886] Page M. J., McKenzie J. E., Bossuyt P. M., Boutron I., Hoffmann T. C., Mulrow C. D., Shamseer L., Tetzlaff J. M., Akl E. A., Brennan S. E., Chou R., Glanville J., Grimshaw J. M., Hróbjartsson A., Lalu M. M., Li T., Loder E. W., Mayo-Wilson E., McDonald S., Moher D. (2021). The PRISMA 2020 statement: An updated guideline for reporting systematic reviews. BMJ.

[B41-behavsci-16-01886] Pulvermüller F., Lutzenberger W., Preissl H. (1999a). Nouns and verbs in the intact brain: Evidence from event-related potentials and high-frequency cortical responses. Cerebral Cortex.

[B42-behavsci-16-01886] Pulvermüller F., Mohr B., Schleichert H. (1999b). Semantic or lexico-syntactic factors: What determines word-class specific activity in the human brain?. Neuroscience Letters.

[B43-behavsci-16-01886] Saccuman M. C., Cappa S. F., Bates E. A., Arevalo A., Della Rosa P., Danna M., Perani D. (2006). The impact of semantic reference on word class: An fMRI study of action and object naming. NeuroImage.

[B44-behavsci-16-01886] Sahin N. T., Pinker S., Halgren E. (2006). Abstract grammatical processing of nouns and verbs in Broca’s area: Evidence from fMRI. Cortex.

[B45-behavsci-16-01886] Shan B. C., Zhang W. T., Ma L., Li D. J., Cao B. L., Tang Y. Y., Wu Y. G., Tang X. W. (2003). Chinese semantic processing cerebral areas. Chinese Science Bulletin.

[B46-behavsci-16-01886] Shapiro K. A., Moo L. R., Caramazza A. (2006). Cortical signatures of noun and verb production. Proceedings of the National Academy of Sciences USA.

[B47-behavsci-16-01886] Shapiro K. A., Mottaghy F. M., Schiller N. O., Poeppel T. D., Fluss M. O., Muller H.-W., Caramazza A., Krause B. J. (2005). Dissociating neural correlates for nouns and verbs. NeuroImage.

[B48-behavsci-16-01886] Shen J. X. (2015a). On subjectivity in Chinese word classes. Foreign Language Teaching and Research.

[B49-behavsci-16-01886] Shen J. X. (2015b). Word class typology and the Chinese nominalism. Contemporary Linguistics.

[B50-behavsci-16-01886] Siri S., Tettamanti M., Cappa S. F., Della Rosa P., Saccuman C., Scifo P., Vigliocco G. (2008). The neural substrate of naming events: Effects of processing demands but not of grammatical class. Cerebral Cortex.

[B51-behavsci-16-01886] Su D. Q., Zhong Y., Zeng H., Ye H. S. (2013). Embodied semantic processing of Chinese action idioms: Evidence from fMRI study. Acta Psychologica Sinica.

[B52-behavsci-16-01886] Sun C. F. (2022). Are there any logical problems with the “noun-verb inclusion” hypothesis?. Journal of Foreign Languages.

[B53-behavsci-16-01886] Tsai P.-S., Lee C.-Y., Tzeng O. J. L., Hung D. L., Yen N.-S. (2009). An event-related potential study of the concreteness effect between Chinese nouns and verbs. Brain Research.

[B54-behavsci-16-01886] Turkeltaub P. E., Eden G. F., Jones K. M., Zeffiro T. A. (2002). Meta-analysis of the functional neuroanatomy of single-word reading: Method and validation. NeuroImage.

[B55-behavsci-16-01886] Tyler L. K., Randall B., Stamatakis E. A. (2008). Cortical differentiation for nouns and verbs depends on grammatical markers. Journal of Cognitive Neuroscience.

[B56-behavsci-16-01886] Vigliocco G., Vinson D. P., Druks J., Barber H., Cappa S. F. (2011). Nouns and verbs in the brain: A review of behavioural, electrophysiological, neuropsychological and imaging studies. Neuroscience & Biobehavioral Reviews.

[B57-behavsci-16-01886] Vigliocco G., Vinson D. P., Siri S., Cappa S. F. (2006). The role of semantics and grammatical class in the neural representation of words. Cerebral Cortex.

[B58-behavsci-16-01886] Wang T., Yang Y. M. (2017). An fMRI study on neural substrates of nouns and verbs in late high-proficient Chinese-English bilinguals. Foreign Language Research.

[B59-behavsci-16-01886] Wang X., Feng S. W., Zhou T. Q., Wang R. Y., Wu G. W., Ni F. S., Yang Y. M. (2021). Brain regions involved in underlying syntactic processing of Mandarin Chinese intransitive verbs: An fMRI study. Brain Sciences.

[B60-behavsci-16-01886] Wang X., Liang D. D., Yang Y. M. (2023). An fMRI study on the comparison of argument syntactic properties of Mandarin intransitive verbs. Contemporary Linguistics.

[B61-behavsci-16-01886] Wang X., Liang D. D., Yang Y. M. (2026a). Differences in the processing of Chinese transitive and intransitive verbs at the behavioral response and neural activity levels. Behavioral Sciences.

[B62-behavsci-16-01886] Wang X., Zhao C., Li X., Liang D. D., Yang Y. M. (2026b). Where does the argument of Chinese unaccusative verbs initially map? An analysis based on the fMRI study. Journal of Psycholinguistic Research.

[B63-behavsci-16-01886] Wu Y. C. (2023). Nouns and verbs. Journal of Foreign Languages.

[B64-behavsci-16-01886] Xi Y. L., Liu L., Hao G., Abudusadike Z., Jiang C. H., Zhang J. R., Wang B. L. (2017). Research of cerebral activation in Uygur-speaking and Chinese-speaking participants during verb generation task with functional magnetic resonance imaging. Medicine.

[B65-behavsci-16-01886] Xi Y. L., Yan W., Kaheman K., Wang J., Jiang C. H., Huang H. X., Wang B. L. (2016). Different brain activation patterns in Uygur and Chinese speakers during verb generation task: A BOLD-fMRI study. International Journal of Clinical and Experimental Medicine.

[B66-behavsci-16-01886] Xia Q. S., Peng G. (2022). The roles of object and action, and concreteness and imageability, in the distinction between nouns and verbs: An ERP study on monosyllabic words in Chinese. Journal of Neurolinguistics.

[B67-behavsci-16-01886] Xia Q. S., Peng G., Shi F. (2014). Hemispheric lateralization in the semantic processing of nouns, verbs and verb-noun ambiguous words in Chinese: Evidence from an ERP study. Journal of Psychological Science.

[B68-behavsci-16-01886] Xia Q. S., Wang L., Peng G. (2016). Nouns and verbs in Chinese are processed differently: Evidence from an ERP study on monosyllabic and disyllabic word processing. Journal of Neurolinguistics.

[B69-behavsci-16-01886] Xia Q. S., Wang T. L., Wang J., Meng Z. Y., Yu M. (2022). To combine is to divide: Evidence of the noun-verb distinction at the morphemic level in compound nouns. Lingua.

[B70-behavsci-16-01886] Yan J., Li W. J., Zhang T. T., Zhang J. J., Jin Z. L., Li L. (2024). Neural mechanism subserving memory of Chinese abstract and concrete words. Journal of University of electronic Science and Technology of China.

[B71-behavsci-16-01886] Yang H., Lin Q., Han Z., Li H., Song L., Chen L., He Y., Bi Y. (2017). Dissociable intrinsic functional networks support noun-object and verb-action processing. Brain and Language.

[B72-behavsci-16-01886] Yang J., Shu H. (2014). Passive reading and motor imagery about hand actions and tool-use actions: An fMRI study. Experimental Brain Research.

[B73-behavsci-16-01886] Yang J., Shu H., Bi Y. C., Liu Y. Y., Wang X. Y. (2011a). Dissociation and association of the embodied representation of tool-use verbs and hand verbs: An fMRI study. Brain and Language.

[B74-behavsci-16-01886] Yang J., Tan L. H., Li P. (2011b). Lexical representation of nouns and verbs in the late bilingual brain. Journal of Neurolinguistics.

[B75-behavsci-16-01886] Yang Y. M., Liang D. D., Gu J. X., Weng X. C., Feng S. W. (2002). A neurolinguistics study on the classification of nouns and verbs in Chinese. Linguistic Sciences.

[B76-behavsci-16-01886] Yu X., Law S. P., Han Z., Zhu C., Bi Y. (2011). Dissociative neural correlates of semantic processing of nouns and verbs in Chinese: A language with minimal inflectional morphology. NeuroImage.

[B77-behavsci-16-01886] Yue Y. (2022). A review of psychological experimental studies on Chinese nouns and verbs. Essays on Linguistics.

[B78-behavsci-16-01886] Zhang W. J., Chen X. M., Wang S. P. (2024). The representation of noun-verb distinction in left posterior middle temporal gyrus: Evidence from representation similarity analyses. Cerebral Cortex.

[B79-behavsci-16-01886] Zhang Y., Wang K., Yue C., Mo N., Wu D., Wen X., Qiu J. (2018). The motor features of action verbs: fMRI evidence using picture naming. Brain and Language.

[B80-behavsci-16-01886] Zhao B., Dang J., Zhang G. (2017). EEG source reconstruction evidence for the noun-verb neural dissociation along semantic dimensions. Neuroscience.

