# Supplementary material for: Neural Representation of Chinese Word Classes: An ALE Meta-Analysis of Noun and Verb Processing
Source: Behav Sci (Basel). 2026 Oct 13;16(10):1886. doi: 10.3390/bs16101886 (PMC13701492; doi:10.3390/bs16101886)
Supplement: Supplementary file 1 [file behavsci-16-01886-s001.zip › behavsci-4464350-supplementary.pdf]

Supplementary File S1 ALE Analysis Inputs and Specifications

Manuscript: Neural Representation of Chinese Word Classes: An ALE Meta-Analysis of Noun and Verb Processing

Purpose. This supplementary file documents the study-to-contrast assignments, coordinate inputs, analysis specifications, and cluster-level results used in the activation likelihood estimation (ALE) workflow. The primary between-set analyses compare baseline-only noun-versus-baseline and verb-versus-baseline datasets. The original mixed noun-associated and verb-associated inputs are retained as additional analyses. This file does not redistribute source articles, participant-level data, software logs, or NIfTI statistical maps.

Table S1. Study-to-contrast assignments

Table S1 records each eligible publication and the contrast contribution coded for the ALE analyses. A value of 1 denotes that the reported contrast was included; it does not denote a participant-independent sample.

study and technique	participants	task	baseline control	N vs B	V vs B	N vs V	V vs N
Klein et al. (1999), PET ($H_2{}^{15}O$-water tracer)	7	Chinese-English verb generation	Word repetition	0	1	0	0
Luke et al. (2002), fMRI	7	Syntactic and semantic plausibility judgment	Font-size judgment	0	1	0	0
Shan et al. (2003), fMRI	9	Chinese semantic processing	Fixation/no-stimulus control	1	0	0	0
Li et al. (2004), fMRI	8	Lexical decision for Chinese nouns and verbs	Fixation	1	1	0	0
Liang et al. (2006), fMRI	6	Visual processing of Chinese noun, verb, and adjective modifiers	Fixation (crosshair)	1	1	0	0
Feng et al. (2007), fMRI	8	Visual processing of Chinese noun, verb, and adjective phrases	Fixation/+ control task	1	0	0	0
Chan et al. (2008), fMRI	11	Noun and verb processing in early bilinguals	Fixation	1	1	0	0
Liu et al. (2009), fMRI	19	Chinese date noun and general noun processing	Rest/fixation +	1	0	0	0
Yang et al. (2011), fMRI	20	Tool-use and hand-action verb processing	Rest	0	1	0	0
Yu et al. (2011), fMRI	21	Semantic processing of Chinese nouns and verbs	Fixation	0	0	1	1
Su et al. (2013), fMRI	13	Semantic comprehension of Chinese action idioms	Symbol baseline conditions	0	1	0	0
Yang et al. (2014), fMRI	20	Passive reading and motor imagery of hand and tool-use actions	Rest	0	1	0	0

Xi et al. (2016), fMRI	15	Verb generation	Initial rest and resting periods	0	1	0	0
Wang et al. (2017), fMRI	18	Noun and verb processing in late high-proficiency Chinese-English bilinguals	Fixation + between task blocks	0	0	0	1
Xi et al. (2017), fMRI	28	Verb generation	Initial rest and resting periods	0	1	0	0
Zhang et al. (2018), fMRI	21	Picture naming of action verbs and nouns	Fixation/ISI implicit baseline	0	0	0	1
Feng et al. (2020), fMRI	17	Syntactic and semantic processing of nouns and verbs in phrases	Arrow congruency judgment	0	0	0	1
Wang et al. (2021), fMRI	16	Syntactic processing of Mandarin Chinese intransitive verbs	Arrow direction judgment control task	0	1	0	0
Wang et al. (2023), fMRI	25	Argument-structure processing of Chinese intransitive verbs	Arrow direction judgment control task	0	1	0	0
Yan et al. (2024), fMRI	28	Chinese concrete and abstract word memory processing	Mini-block interval/rest	1	0	0	0
Zhang et al. (2024), fMRI	20	Noun-verb distinction in left posterior middle temporal gyrus	Passive viewing of an asterisk	1	1	0	0
Wang et al. (2026a), fMRI	25	Processing of Chinese transitive and intransitive verbs	Arrow direction judgment control task	0	1	0	0
Wang et al. (2026b), fMRI	25	Argument mapping of Chinese unaccusative verbs	Arrow direction judgment control task	0	1	0	0

Table S2. ALE analysis specifications

All analyses were conducted in GingerALE 3.0.2 in MNI space, using the gray-matter mask specified in the saved outputs and 1,000 permutations. Table S2 distinguishes the primary baseline-only workflow from the additional mixed-input workflow.

Analysis	Input	Inference and threshold
N>B ALE map	8 datasets; 132 foci; 109 participant entries	Cluster-level FWE p = .05; uncorrected cluster-forming p = .05; 1,000 permutations.
V>B ALE map	15 datasets; 282 foci; 246 participant entries	Cluster-level FWE p = .05; uncorrected cluster-forming p = .05; 1,000 permutations.
Primary baseline-only pooled reference	23 datasets; 414 foci; 355 participant entries	Cluster-level FWE p = .05; uncorrected cluster-forming p = .05; 1,000 permutations.
Primary N>B > V>B and V>B > N>B	8 N>B versus 15 V>B datasets	Uncorrected voxel-level p = .05; minimum cluster volume 200 mm^3; 1,000 permutations.

Analysis	Input	Inference and threshold
Primary baseline-only conjunction	N>B, V>B, and 23-entry pooled reference maps	Performed within the baseline-only comparison/conjunction workflow; no suprathreshold cluster.
Additional mixed inputs A and B	A = 8 N>B + 1 N>V (9 datasets; 140 foci); B = 15 V>B + 4 V>N (19 datasets; 316 foci)	Cluster-level FWE p = .05; uncorrected cluster-forming p = .05; 1,000 permutations.
Additional mixed pooled reference	28 datasets; 456 foci; 453 participant entries	Cluster-level FWE p = .05; uncorrected cluster-forming p = .05; 1,000 permutations.
Additional A>B and B>A	Mixed A and B inputs	Uncorrected voxel-level p = .05; minimum cluster volume 200 mm³; 1,000 permutations.
Additional mixed-input conjunction	A, B, and 28-entry pooled reference maps	Performed within the mixed-input comparison/conjunction workflow; no suprathreshold cluster.

Table S3. ALE analysis results

The exact N>B and V>B inputs are reproduced in Data S1 and Data S2. Their union forms the 23-entry baseline-only pooled reference. Data S3 and Data S4 reproduce the additional mixed inputs A and B, and Data S5 reproduces their 28-entry pooled reference. Comment lines identify the reference space, source study, contrast, and reported subject count; subsequent tab-separated rows contain x, y, and z coordinates in MNI space.

Analysis	Cluster	Volume (mm³)	Peak MNI coordinate and Z	Principal anatomical location
Primary N>B > V>B (total 5,072 mm³)	1	4784	-46, -36.5, 46.3; Z = 3.29	Left inferior parietal lobule, postcentral and supramarginal gyri (BA40, BA2, BA3)
Primary N>B > V>B	2	288	31, 28.3, -0.3; Z = 2.07	Right claustrum/insula (BA13)
Primary V>B > N>B (total 7,272 mm³)	1	3560	-37.7, 6.3, 33.2; Z = 3.29	Left precentral, inferior frontal, and middle frontal gyri (BA6, BA9, BA46)
Primary V>B > N>B	2	2192	36, -78, -14; Z = 3.29	Right occipital/posterior cerebellar region, including fusiform and occipital gyri and declive (BA18, BA19)
Primary V>B > N>B	3	1520	54.9, 5.7, 40.6; Z = 3.09	Right precentral, middle frontal, and inferior frontal gyri (BA6, BA9)
Primary baseline-only conjunction	--	--	--	No suprathreshold cluster
Additional A>B (total 5,624 mm³)	1	4536	-45.9, -34.9, 46; Z = 2.75	Left inferior parietal lobule, postcentral and supramarginal gyri (BA40, BA2)
Additional A>B	2	368	-50, -68, -20; Z = 2.51	Left cerebellar declive and fusiform gyrus (BA37)
Additional A>B	3	264	-36.8, -7.1, 56.3; Z = 1.85	Left precentral gyrus (BA6, BA4)
Additional A>B	4	240	30, 26, -2; Z = 2.05	Right claustrum/insula (BA13)
Additional A>B	5	216	-56, 21, 11; Z = 2.10	Left inferior frontal gyrus (BA44, BA45)
Additional B>A (total 7,104 mm³)	1	3184	-39.2, 9.6, 32; Z = 3.29	Left precentral, inferior frontal, and middle frontal gyri (BA6, BA9)
Additional B>A	2	1664	52, -8, 40; Z = 2.75	Right precentral, middle

Analysis	Cluster	Volume (mm³)	Peak MNI coordinate and Z	Principal anatomical location
				frontal, and inferior frontal gyri (BA6, BA9, BA4)
Additional B>A	3	1152	36, -79.3, -16; Z = 2.29	Right occipital/posterior cerebellar region, including fusiform and occipital gyri and declive (BA18, BA19)
Additional B>A	4	888	8, -88, -18; Z = 1.96	Predominantly right posterior cerebellum, including declive and pyramis
Additional B>A	5	216	-4, 16, 60; Z = 2.03	Left superior frontal gyrus (BA6)
Additional mixed-input conjunction	--	--	--	No suprathreshold cluster

Note. N>B = noun versus baseline; V>B = verb versus baseline. A = noun-associated mixed input containing 8 N>B datasets and 1 N>V dataset. B = verb-associated mixed input containing 15 V>B datasets and 4 V>N datasets. Peak Z denotes the highest positive Z value reported for each saved cluster. Anatomical descriptions reproduce the principal labels in the saved GingerALE cluster outputs.

Section S1. Coordinate input files

Coordinates are reproduced exactly as submitted to the ALE workflow. Comment lines identify the reference space, source study, contrast, and reported subject count; subsequent tab-separated rows contain x, y, and z coordinates in MNI space.

Data S1. Noun-versus-baseline coordinate input

8 experiment-level datasets; 132 foci.

```
// Reference=MNI
// Yan, 2024: Noun > Baseline
// Subjects=28
-33	-84	-12
-21	-90	-12
-39	-60	-18
24	-84	-9
42	-60	-12
39	-54	-18
-12	48	42
-6	42	48
-12	21	63
-42	30	-15
-54	27	15
-51	33	3
-39	-63	21
51	27	12
51	27	-6
51	39	9
-54	-6	-12

// Zhang, 2024: Noun > Baseline
// Subjects=20
42	-58	31
-12	-10	8
-27	26	2
27	26	2
-42	-40	44
33	-55	50

// Shan, 2003: Noun > Baseline
// Subjects=9
-55.03	17.4	8.08
-55.29	32.96	-9.15
-48.03	-35.6	37.85
72.62	-18.11	11.66
-48.7	-46.11	-17.12
-57.04	-23.03	12.11
74.75	-18.31	9.4
-22.24	-102.23	12.63
25.24	-102.48	7.37
-46.54	-69.06	-21.61
48.53	-68.73	-23.26

// Li, 2004: Noun > Baseline
```

```
// Subjects=8
-52.75	15.04	17.24
-50.77	32.3	6.53
-50.1	-18.51	48.52
-37.04	-9.02	57.45
46.66	0.19	13.65
31.17	17.1	-11.29
37.72	28.59	-3.58
69.44	-15.47	17.05
-54.12	9.56	-6.85
48.57	-7.19	-8.06
-45.75	-29.24	48.39
-32.69	-40.48	53.76
-52.84	-68.8	-6.96
-50.77	-62.93	-13.18
-15.72	-98.6	16.64
-11.44	15.99	36.61
16.64	18.22	35.91
5.6	-4.41	12.56
27.13	-4.86	6.64
18.65	-5.87	19.21
-22.74	-29.34	-11.38
-31.45	14.96	-7.77
13.99	-69.71	-20.34

// Chan, 2008: Noun > Baseline
// Subjects=11
-47.21	23.75	29.73
-33.5	44.27	7.29
-42.03	26.47	12.56
-41.86	-1.83	19.84
-2.53	5.99	55.38
-33.52	12.39	-.76
-35.02	-5.7	47
53.23	27.09	25.44
49.91	23.27	19.16
41.98	11.72	-12.06
-45.67	8.14	-22.54
-44.69	-45.81	43.29
-25.16	-63.72	45.85
-22.4	-95.72	1.91
-52.98	-58.89	-15.79
30.92	-64.25	37.1
-33.7	-81.96	-34
39.81	-56.2	-26.6

// Liang, 2006: Noun > Baseline
// Subjects=6
27.66	77.4	-20.56
48.92	-35.21	13.75
8.72	17.72	8.08
35.67	26.12	4.55
-29.75	95.83	-27.01
-38.41	62.13	49.31
-32.75	31.28	63.47
-6.4	13.31	7.65
0.85	3.74	65.63
8.87	-45.75	6.51

// Feng, 2007: Noun > Baseline
// Subjects=8
-2.86	14.44	31.01
-4.42	57.89	2.09
-46.83	31.64	-23.73
-52.39	7.86	43.71
-62.36	-54.2	11.92
-28.33	-46.74	55.43
-28.85	-85.65	6.62
-1.2	54.5	0.13

// Liu, 2009: Noun > Baseline
// Subjects=19
-38.18	-40.05	47.09
-27.34	-52.88	47.06
-49.08	-33.17	40.99
-41.9	-67.72	3.95
-46.22	-84.21	0.05
-42.31	-55.53	-26.38
43.38	-67.94	-3.08
46.44	-69.28	-17.57
46.35	-59.06	-23.06
-2.63	13.98	48.98
-2.42	4.59	63.36
-52.44	6.38	39.38
-31.58	0.23	64.28
-38.11	-.21	59.96
-32.19	-103.93	-4.96
-41.96	-88.97	-5.15
-42.24	-77.97	-25.29
-39.1	-67.74	-30.83
25.65	8.94	62.45
39.66	4.32	58.18
29.27	-104.65	-16.02
```

43.15	-69.71	-21.96
38.96	-92.81	-16.24
-49.04	-25.2	45.81
-6.95	10.67	48.26
-20.79	-55.64	51.7
-35.39	-95.98	-0.09
-49.28	-23.78	26.62
-42.2	-70.01	-20.47
-35.65	-90.3	-19.7
22.41	8.93	62.5
4.99	14.42	53.29
53.17	11.37	18.04
33.11	-53.09	41.56
50.81	-61.3	-12.83
53.67	-24.32	49.57
47.35	-38.4	60.04
46.63	-88.66	-6.69
36.89	-100.91	-10.92

Data S2. Verb-versus-baseline coordinate input

15 experiment-level datasets; 282 foci.

// Reference=MNI
// Klein, 1999: Verb > Baseline
// Subjects=7
-46 34 8
-46 27 23
-44 18 33
0 24 47
-52 -40 -2
-27 -62 47
4 -87 11
36 -69 -23
7 -83 -21
9 30 27

// Xi, 2016: Verb > Baseline
// Subjects=15
-33 -87 -18
-21 -90 -18
-36 -90 3
-18 60 9
-27 57 9
-33 54 3
-30 15 30
-48 6 45
-39 6 39
36 -69 -24
33 -75 -30
33 -60 -30
-9 0 66
0 9 63
6 6 72
-24 -45 45
-30 -63 66
-27 -57 51

// Xi, 2017: Verb > Baseline
// Subjects=28
-15 -3 21
-30 -87 -9
-30 -81 -18
-36 -60 -21
-3 15 51
-12 15 36
3 6 66
-27 -63 39
-30 -60 51
-30 -54 36
-42 -45 42
-39 -39 33
51 9 33
33 24 9
48 12 3
9 -84 -24
33 -78 -27
21 -84 -24

// Wang, 2021: Verb > Baseline
// Subjects=16
-23 -93 -7
-42 -60 -21
-46 9 25
-53 16 35
-57 -48 11
-8 8 53
-27 -56 42
-5 -71 28
26 -90 -7
44 -60 -21
41 -67 -1
52 16 32
7 -75 -28
-23 -90 -7
-46 -60 -11
-46 4 32
-46 23 25
-35 1 42
-8 4 56
-27 56 42
-53 -48 11
-8 -75 -28
29 -90 -7
52 16 32
7 -75 -28
-5 -56 -18
-27 -90 -11
-53 12 35

-42 19 18
-27 -56 42
-5 8 53
29 -90 -7
52 16 35
33 -48 -28

// Zhang, 2024: Verb > Baseline
// Subjects=20
12 -73 -25
12 -19 14
-36 5 44

// Wang, 2026: Verb > Baseline
// Subjects=25
-55 0 -42
-48 21 18
-38 25 14
-38 -53 -11
-6 11 56
54 -11 53
47 -21 60
36 -81 -11
29 -84 -4
-48 25 21
-45 7 28
-52 4 46
-3 14 53
-10 7 70
-27 -63 42
-48 -60 -14
-6 -70 7
5 25 39
40 -74 -14
29 -84 -4
54 35 25
36 28 11
29 -60 39

// Wang, 2026: Verb > Baseline
// Subjects=25
-48 28 5
-41 18 21
-45 11 28
-27 -88 -7
-41 -49 -18
-41 -46 -7
36 -88 -7
43 -70 -14
40 -18 63
29 -21 70
-27 -88 -7
-41 -49 -18
-48 28 14
-6 4 70
-3 14 56
-3 -63 -14
36 -88 -7
43 -60 -18
50 -14 56
36 -35 67
57 32 21
57 21 32
-34 -88 -7
-41 -49 -18
-41 -67 -14
-48 28 4
-41 18 21
-55 -11 18
47 -17 60

// Wang, 2023: Verb > Baseline
// Subjects=25
-41 -49 18
-45 -60 -14
-31 -32 -4
-52 4 46
-41 4 32
-48 14 -14
-3 -53 7
-55 -35 4
50 -14 56
29 -81 -4
36 -74 -7
33 -39 0
-41 -53 -18
-48 14 -14
-55 -4 -7
-3 7 63
-34 28 11
33 -74 -7
54 -11 53
29 -25 70

57	11	-7
20	-18	4

// Su, 2013: Verb > Baseline
// Subjects=13
45	-78	-6
33	-81	-12
-18	-93	-6
-9	72	42
9	-66	42
-45	54	51
-42	-45	57
51	-42	39
42	-54	57
-57	-30	45
54	12	9
42	3	3
0	-30	27
0	-27	45
-33	24	45
-48	0	54
-51	3	45
-57	-27	-24
-54	-39	-15
-39	-45	-24

// Luke, 2002: Verb > Baseline
// Subjects=7
-47.31	31.74	23.33
-47.51	47.72	10.55
-59.73	38.6	-17.47
-52.63	14.81	26.22
-7.94	73.33	-16.19
-1.48	27.23	-27.43
-11.05	3.15	-20.4
36.4	62.86	-15.91
60.59	30.02	10.46
-47.26	26.63	26.08
-66.02	-9.18	-11.52
47.1	15.08	-33.77
-21.35	-102.52	-1.92
33.63	-92.31	-10.59
-28.35	-52.81	-32.49
48.39	-47.87	-29.81
4.27	-83.98	-23.24
-49.4	24.6	27.44
-47.61	38.28	1.4
-52.63	14.81	26.22
0.59	42.26	52.85
-18.76	64.37	-19.6
-7.1	51.81	-39.85
64.71	46.02	-2.4
37.42	58.09	-21.06
60.87	25.51	31.07
-54.3	19.16	-19
-64.78	-28.76	-2.88
-49.32	-60.12	16.77
70.25	-13.08	-3.37
54.61	16.92	-37.45
-23.58	-101.98	-7.54
32.55	-93.38	-10.47
-3.06	-12.2	10.12
12.1	-17.36	11.49
-11.02	9.95	-16.59
-36.98	-33.08	-27.58
-30.5	-59.3	-32.94
46.28	-51.93	-27.13
13.87	-76.06	-30.91

// Li, 2004: Verb > Baseline
// Subjects=8
-52.96	36.44	5.04
-52.26	-.98	52.43
-53.48	-5.32	40.55
-40.1	17.06	-7.83
51.57	-6.38	57.92
39.79	16.93	-13.66
10.47	15.06	59.86
60.53	19.72	3.64
53.17	15.73	18.73
-42.43	-6.09	-25.67
40.59	-31.79	39.33
-50.66	-69.75	-5.79
-26.36	-63.49	36.88
-40.11	-30.02	-17.74
-17.95	-94.77	11.82
29.31	-86.8	-8.82
-18.24	-13.73	6.05
-42.29	-58.62	-24.96
29.06	-61.35	-23.66

// Chan, 2008: Verb > Baseline
// Subjects=11

-45.12	25.37	23.93
-41.86	4.76	21.43
-43.25	36.37	3.76
-18.61	17.37	-17.19
-1.5	22.05	54.89
-47.66	11.61	-8.28
-27.4	-3.02	52.21
44.41	39.95	14.24
45.44	28.7	8.61
2.69	27.61	45.3
42.6	-0.08	33.92
-39.18	-51.55	50.49
-34.81	-42.4	56.23
-24.03	-65.53	49.37
-47.5	-55.06	-9.53
4.74	29.02	37.28
-7.41	-22.31	5.59
-33.7	-81.96	-34
5.41	-78.04	-17.13

// Yang, 2011: Verb > Baseline
// Subjects=20

-28.02	-86.46	-13.48
-47.09	8.22	35.74
-49.69	39.96	7.99
-27.31	-54.91	48.38
54.58	9.4	42.86
47.58	-73.22	-13.84
2.9	12.8	59.09
29.79	-59.59	-50.74

// Yang, 2014: Verb > Baseline
// Subjects=20

-36.34	-.88	29.74
-38.9	7.05	-.14
-1.55	9.62	48.28
-55.78	-38.15	22.55
-41.22	-46.71	-23.91
-32.29	-86.06	-8.97
54.5	0.07	34.82
38.85	24.55	-.95
21.77	-87.78	-7.48
32.25	-57.29	-26.36
27.61	-61.93	-52.72

// Liang, 2006: Verb > Baseline
// Subjects=6

29.82	79.64	-19.7
24.18	68.42	44.26
34.65	-29.27	-2.28
52.23	-9.49	24.6
-36.13	73.57	-23.58
24.18	68.42	44.26
-46.92	28.06	52.83
7.19	3.79	54.31

Data S3. Additional noun-associated mixed input (A)

8 N>B datasets plus 1 original N>V dataset; 9 datasets and 140 foci.

// Reference=MNI
// Yan, 2024: Noun > Baseline
// Subjects=28

-33	-84	-12
-21	-90	-12
-39	-60	-18
24	-84	-9
42	-60	-12
39	-54	-18
-12	48	42
-6	42	48
-12	21	63
-42	30	-15
-54	27	15
-51	33	3
-39	-63	21
51	27	12
51	27	-6
51	39	9
-54	-6	-12

// Zhang, 2024: Noun > Baseline
// Subjects=20

42	-58	31
-12	-10	8
-27	26	2
27	26	2
-42	-40	44
33	-55	50

// Shan, 2003: Noun > Baseline
// Subjects=9

-55.03	17.4	8.08
-55.29	32.96	-9.15
-48.03	-35.6	37.85
72.62	-18.11	11.66
-48.7	-46.11	-17.12
-57.04	-23.03	12.11
74.75	-18.31	9.4
-22.24	-102.23	12.63
25.24	-102.48	7.37
-46.54	-69.06	-21.61
48.53	-68.73	-23.26

// Li, 2004: Noun > Baseline
// Subjects=8

-52.75	15.04	17.24
-50.77	32.3	6.53
-50.1	-18.51	48.52
-37.04	-9.02	57.45
46.66	0.19	13.65
31.17	17.1	-11.29
37.72	28.59	-3.58
69.44	-15.47	17.05
-54.12	9.56	-6.85
48.57	-7.19	-8.06
-45.75	-29.24	48.39
-32.69	-40.48	53.76
-52.84	-68.8	-6.96
-50.77	-62.93	-13.18
-15.72	-98.6	16.64
-11.44	15.99	36.61
16.64	18.22	35.91
5.6	-4.41	12.56
27.13	-4.86	6.64
18.65	-5.87	19.21
-22.74	-29.34	-11.38
-31.45	14.96	-7.77
13.99	-69.71	-20.34

// Chan, 2008: Noun > Baseline
// Subjects=11

-47.21	23.75	29.73
-33.5	44.27	7.29
-42.03	26.47	12.56
-41.86	-1.83	19.84
-2.53	5.99	55.38
-33.52	12.39	-.76
-35.02	-5.7	47
53.23	27.09	25.44
49.91	23.27	19.16
41.98	11.72	-12.06
-45.67	8.14	-22.54
-44.69	-45.81	43.29
-25.16	-63.72	45.85
-22.4	-95.72	1.91
-52.98	-58.89	-15.79

30.92	-64.25	37.1
-33.7	-81.96	-34
39.81	-56.2	-26.6

// Liang, 2006: Noun > Baseline

// Subjects=6

27.66	77.4	-20.56
48.92	-35.21	13.75
8.72	17.72	8.08
35.67	26.12	4.55
-29.75	95.83	-27.01
-38.41	62.13	49.31
-32.75	31.28	63.47
-6.4	13.31	7.65
0.85	3.74	65.63
8.87	-45.75	6.51

// Feng, 2007: Noun > Baseline

// Subjects=8

-2.86	14.44	31.01
-4.42	57.89	2.09
-46.83	31.64	-23.73
-52.39	7.86	43.71
-62.36	-54.2	11.92
-28.33	-46.74	55.43
-28.85	-85.65	6.62
-1.2	54.5	0.13

// Liu, 2009: Noun > Baseline

// Subjects=19

-38.18	-40.05	47.09
-27.34	-52.88	47.06
-49.08	-33.17	40.99
-41.9	-67.72	3.95
-46.22	-84.21	0.05
-42.31	-55.53	-26.38
43.38	-67.94	-3.08
46.44	-69.28	-17.57
46.35	-59.06	-23.06
-2.63	13.98	48.98
-2.42	4.59	63.36
-52.44	6.38	39.38
-31.58	0.23	64.28
-38.11	-.21	59.96
-32.19	-103.93	-4.96
-41.96	-88.97	-5.15
-42.24	-77.97	-25.29
-39.1	-67.74	-30.83
25.65	8.94	62.45
39.66	4.32	58.18
29.27	-104.65	-16.02
43.15	-69.71	-21.96
38.96	-92.81	-16.24
-49.04	-25.2	45.81
-6.95	10.67	48.26
-20.79	-55.64	51.7
-35.39	-95.98	-0.09
-49.28	-23.78	26.62
-42.2	-70.01	-20.47
-35.65	-90.3	-19.7
22.41	8.93	62.5
4.99	14.42	53.29
53.17	11.37	18.04
33.11	-53.09	41.56
50.81	-61.3	-12.83
53.67	-24.32	49.57
47.35	-38.4	60.04
46.63	-88.66	-6.69
36.89	-100.91	-10.92

// Yu, 2011: Noun > Verb

// Subjects=21

-42	33	-18
-12	42	42
-3	36	-24
-54	3	-33
-30	-33	-21
33	-30	-21
-33	-72	39
45	-75	45

Data S4. Additional verb-associated mixed input (B)

15 V>B datasets plus 4 original V>N datasets; 19 datasets and 316 foci.

// Reference=MNI
// Klein, 1999: Verb > Baseline
// Subjects=7

-46	34	8
-46	27	23
-44	18	33
0	24	47
-52	-40	-2
-27	-62	47
4	-87	11
36	-69	-23
7	-83	-21
9	30	27

// Xi, 2016: Verb > Baseline
// Subjects=15

-33	-87	-18
-21	-90	-18
-36	-90	3
-18	60	9
-27	57	9
-33	54	3
-30	15	30
-48	6	45
-39	6	39
36	-69	-24
33	-75	-30
33	-60	-30
-9	0	66
0	9	63
6	6	72
-24	-45	45
-30	-63	66
-27	-57	51

// Xi, 2017: Verb > Baseline
// Subjects=28

-15	-3	21
-30	-87	-9
-30	-81	-18
-36	-60	-21
-3	15	51
-12	15	36
3	6	66
-27	-63	39
-30	-60	51
-30	-54	36
-42	-45	42
-39	-39	33
51	9	33
33	24	9
48	12	3
9	-84	-24
33	-78	-27
21	-84	-24

// Wang, 2021: Verb > Baseline
// Subjects=16

-23	-93	-7
-42	-60	-21
-46	9	25
-53	16	35
-57	-48	11
-8	8	53
-27	-56	42
-5	-71	28
26	-90	-7
44	-60	-21
41	-67	-1
52	16	32
7	-75	-28
-23	-90	-7
-46	-60	-11
-46	4	32
-46	23	25
-35	1	42
-8	4	56
-27	56	42
-53	-48	11
-8	-75	-28
29	-90	-7
52	16	32
7	-75	-28
-5	-56	-18
-27	-90	-11
-53	12	35
-42	19	18

-27	-56	42
-5	8	53
29	-90	-7
52	16	35
33	-48	-28

// Zhang, 2024: Verb > Baseline
// Subjects=20

12	-73	-25
12	-19	14
-36	5	44

// Wang, 2026: Verb > Baseline
// Subjects=25

-55	0	-42
-48	21	18
-38	25	14
-38	-53	-11
-6	11	56
54	-11	53
47	-21	60
36	-81	-11
29	-84	-4
-48	25	21
-45	7	28
-52	4	46
-3	14	53
-10	7	70
-27	-63	42
-48	-60	-14
-6	-70	7
5	25	39
40	-74	-14
29	-84	-4
54	35	25
36	28	11
29	-60	39

// Wang, 2026: Verb > Baseline
// Subjects=25

-48	28	5
-41	18	21
-45	11	28
-27	-88	-7
-41	-49	-18
-41	-46	-7
36	-88	-7
43	-70	-14
40	-18	63
29	-21	70
-27	-88	-7
-41	-49	-18
-48	28	14
-6	4	70
-3	14	56
-3	-63	-14
36	-88	-7
43	-60	-18
50	-14	56
36	-35	67
57	32	21
57	21	32
-34	-88	-7
-41	-49	-18
-41	-67	-14
-48	28	4
-41	18	21
-55	-11	18
47	-17	60

// Wang, 2023: Verb > Baseline
// Subjects=25

-41	-49	18
-45	-60	-14
-31	-32	-4
-52	4	46
-41	4	32
-48	14	-14
-3	-53	7
-55	-35	4
50	-14	56
29	-81	-4
36	-74	-7
33	-39	0
-41	-53	-18
-48	14	-14
-55	-4	-7
-3	7	63
-34	28	11
33	-74	-7
54	-11	53
29	-25	70
57	11	-7

20	-18	4

// Su, 2013: Verb > Baseline
// Subjects=13

45	-78	-6
33	-81	-12
-18	-93	-6
-9	72	42
9	-66	42
-45	54	51
-42	-45	57
51	-42	39
42	-54	57
-57	-30	45
54	12	9
42	3	3
0	-30	27
0	-27	45
-33	24	45
-48	0	54
-51	3	45
-57	-27	-24
-54	-39	-15
-39	-45	-24

// Luke, 2002: Verb > Baseline
// Subjects=7

-47.31	31.74	23.33
-47.51	47.72	10.55
-59.73	38.6	-17.47
-52.63	14.81	26.22
-7.94	73.33	-16.19
-1.48	27.23	-27.43
-11.05	3.15	-20.4
36.4	62.86	-15.91
60.59	30.02	10.46
-47.26	26.63	26.08
-66.02	-9.18	-11.52
47.1	15.08	-33.77
-21.35	-102.52	-1.92
33.63	-92.31	-10.59
-28.35	-52.81	-32.49
48.39	-47.87	-29.81
4.27	-83.98	-23.24
-49.4	24.6	27.44
-47.61	38.28	1.4
-52.63	14.81	26.22
0.59	42.26	52.85
-18.76	64.37	-19.6
-7.1	51.81	-39.85
64.71	46.02	-2.4
37.42	58.09	-21.06
60.87	25.51	31.07
-54.3	19.16	-19
-64.78	-28.76	-2.88
-49.32	-60.12	16.77
70.25	-13.08	-3.37
54.61	16.92	-37.45
-23.58	-101.98	-7.54
32.55	-93.38	-10.47
-3.06	-12.2	10.12
12.1	-17.36	11.49
-11.02	9.95	-16.59
-36.98	-33.08	-27.58
-30.5	-59.3	-32.94
46.28	-51.93	-27.13
13.87	-76.06	-30.91

// Li, 2004: Verb > Baseline
// Subjects=8

-52.96	36.44	5.04
-52.26	-.98	52.43
-53.48	-5.32	40.55
-40.1	17.06	-7.83
51.57	-6.38	57.92
39.79	16.93	-13.66
10.47	15.06	59.86
60.53	19.72	3.64
53.17	15.73	18.73
-42.43	-6.09	-25.67
40.59	-31.79	39.33
-50.66	-69.75	-5.79
-26.36	-63.49	36.88
-40.11	-30.02	-17.74
-17.95	-94.77	11.82
29.31	-86.8	-8.82
-18.24	-13.73	6.05
-42.29	-58.62	-24.96
29.06	-61.35	-23.66

// Chan, 2008: Verb > Baseline
// Subjects=11

-45.12	25.37	23.93

-41.86 4.76 21.43
-43.25 36.37 3.76
-18.61 17.37 -17.19
-1.5 22.05 54.89
-47.66 11.61 -8.28
-27.4 -3.02 52.21
44.41 39.95 14.24
45.44 28.7 8.61
2.69 27.61 45.3
42.6 -0.08 33.92
-39.18 -51.55 50.49
-34.81 -42.4 56.23
-24.03 -65.53 49.37
-47.5 -55.06 -9.53
4.74 29.02 37.28
-7.41 -22.31 5.59
-33.7 -81.96 -34
5.41 -78.04 -17.13

// Yang, 2011: Verb > Baseline
// Subjects=20
-28.02 -86.46 -13.48
-47.09 8.22 35.74
-49.69 39.96 7.99
-27.31 -54.91 48.38
54.58 9.4 42.86
47.58 -73.22 -13.84
2.9 12.8 59.09
29.79 -59.59 -50.74

// Yang, 2014: Verb > Baseline
// Subjects=20
-36.34 -.88 29.74
-38.9 7.05 -.14
-1.55 9.62 48.28
-55.78 -38.15 22.55
-41.22 -46.71 -23.91
-32.29 -86.06 -8.97
54.5 0.07 34.82
38.85 24.55 -.95
21.77 -87.78 -7.48
32.25 -57.29 -26.36
27.61 -61.93 -52.72

// Liang, 2006: Verb > Baseline
// Subjects=6
29.82 79.64 -19.7
24.18 68.42 44.26
34.65 -29.27 -2.28
52.23 -9.49 24.6
-36.13 73.57 -23.58
24.18 68.42 44.26
-46.92 28.06 52.83
7.19 3.79 54.31

// Yu, 2011: Verb > Noun
// Subjects=21
-51 9 6
-45 -51 9
51 -39 9
21 -63 9
24 -69 -48

// Zhang, 2018: Verb > Noun
// Subjects=21
-38 -22 50
52 -6 42
-6 -8 54
-36 -24 12
-14 -20 2
62 6 -8
68 -4 18
-54 -8 4
-64 -16 8

// Feng, 2020: Verb > Noun
// Subjects=17
-50 38 24
-26 -7 -7
-16 -79 -16
18 -81 -14
-32 40 10
30 46 8
-3 23 54
-42 15 41
-40 20 35
36 -20 -30
-49 42 29
-47 -52 -17
-44 41 5
-46 -37 -17

// Wang, 2017: Verb > Noun
// Subjects=18

54	-63	3
48	-51	3
-39	-78	15
-48	12	12
-51	-63	3
36	-81	27

Data S5. Additional mixed pooled coordinate input

All 28 experiment-level datasets; 456 foci.

// Reference=MNI
// Yan, 2024: Noun > Baseline
// Subjects=28

-33	-84	-12
-21	-90	-12
-39	-60	-18
24	-84	-9
42	-60	-12
39	-54	-18
-12	48	42
-6	42	48
-12	21	63
-42	30	-15
-54	27	15
-51	33	3
-39	-63	21
51	27	12
51	27	-6
51	39	9
-54	-6	-12

// Zhang, 2024: Noun > Baseline
// Subjects=20

42	-58	31
-12	-10	8
-27	26	2
27	26	2
-42	-40	44
33	-55	50

// Shan, 2003: Noun > Baseline
// Subjects=9

-55.03	17.4	8.08
-55.29	32.96	-9.15
-48.03	-35.6	37.85
72.62	-18.11	11.66
-48.7	-46.11	-17.12
-57.04	-23.03	12.11
74.75	-18.31	9.4
-22.24	-102.23	12.63
25.24	-102.48	7.37
-46.54	-69.06	-21.61
48.53	-68.73	-23.26

// Li, 2004: Noun > Baseline
// Subjects=8

-52.75	15.04	17.24
-50.77	32.3	6.53
-50.1	-18.51	48.52
-37.04	-9.02	57.45
46.66	0.19	13.65
31.17	17.1	-11.29
37.72	28.59	-3.58
69.44	-15.47	17.05
-54.12	9.56	-6.85
48.57	-7.19	-8.06
-45.75	-29.24	48.39
-32.69	-40.48	53.76
-52.84	-68.8	-6.96
-50.77	-62.93	-13.18
-15.72	-98.6	16.64
-11.44	15.99	36.61
16.64	18.22	35.91
5.6	-4.41	12.56
27.13	-4.86	6.64
18.65	-5.87	19.21
-22.74	-29.34	-11.38
-31.45	14.96	-7.77
13.99	-69.71	-20.34

// Chan, 2008: Noun > Baseline
// Subjects=11

-47.21	23.75	29.73
-33.5	44.27	7.29
-42.03	26.47	12.56
-41.86	-1.83	19.84
-2.53	5.99	55.38
-33.52	12.39	-.76
-35.02	-5.7	47
53.23	27.09	25.44
49.91	23.27	19.16
41.98	11.72	-12.06
-45.67	8.14	-22.54
-44.69	-45.81	43.29
-25.16	-63.72	45.85
-22.4	-95.72	1.91
-52.98	-58.89	-15.79

30.92 -64.25 37.1
-33.7 -81.96 -34
39.81 -56.2 -26.6

// Liang, 2006: Noun > Baseline
// Subjects=6
27.66 77.4 -20.56
48.92 -35.21 13.75
8.72 17.72 8.08
35.67 26.12 4.55
-29.75 95.83 -27.01
-38.41 62.13 49.31
-32.75 31.28 63.47
-6.4 13.31 7.65
0.85 3.74 65.63
8.87 -45.75 6.51

// Feng, 2007: Noun > Baseline
// Subjects=8
-2.86 14.44 31.01
-4.42 57.89 2.09
-46.83 31.64 -23.73
-52.39 7.86 43.71
-62.36 -54.2 11.92
-28.33 -46.74 55.43
-28.85 -85.65 6.62
-1.2 54.5 0.13

// Liu, 2009: Noun > Baseline
// Subjects=19
-38.18 -40.05 47.09
-27.34 -52.88 47.06
-49.08 -33.17 40.99
-41.9 -67.72 3.95
-46.22 -84.21 0.05
-42.31 -55.53 -26.38
43.38 -67.94 -3.08
46.44 -69.28 -17.57
46.35 -59.06 -23.06
-2.63 13.98 48.98
-2.42 4.59 63.36
-52.44 6.38 39.38
-31.58 0.23 64.28
-38.11 -.21 59.96
-32.19 -103.93 -4.96
-41.96 -88.97 -5.15
-42.24 -77.97 -25.29
-39.1 -67.74 -30.83
25.65 8.94 62.45
39.66 4.32 58.18
29.27 -104.65 -16.02
43.15 -69.71 -21.96
38.96 -92.81 -16.24
-49.04 -25.2 45.81
-6.95 10.67 48.26
-20.79 -55.64 51.7
-35.39 -95.98 -0.09
-49.28 -23.78 26.62
-42.2 -70.01 -20.47
-35.65 -90.3 -19.7
22.41 8.93 62.5
4.99 14.42 53.29
53.17 11.37 18.04
33.11 -53.09 41.56
50.81 -61.3 -12.83
53.67 -24.32 49.57
47.35 -38.4 60.04
46.63 -88.66 -6.69
36.89 -100.91 -10.92

// Yu, 2011: Noun > Verb
// Subjects=21
-42 33 -18
-12 42 42
-3 36 -24
-54 3 -33
-30 -33 -21
33 -30 -21
-33 -72 39
45 -75 45

// Klein, 1999: Verb > Baseline
// Subjects=7
-46 34 8
-46 27 23
-44 18 33
0 24 47
-52 -40 -2
-27 -62 47
4 -87 11
36 -69 -23
7 -83 -21

9	30	27

// Xi, 2016: Verb > Baseline
// Subjects=15
-33	-87	-18
-21	-90	-18
-36	-90	3
-18	60	9
-27	57	9
-33	54	3
-30	15	30
-48	6	45
-39	6	39
36	-69	-24
33	-75	-30
33	-60	-30
-9	0	66
0	9	63
6	6	72
-24	-45	45
-30	-63	66
-27	-57	51

// Xi, 2017: Verb > Baseline
// Subjects=28
-15	-3	21
-30	-87	-9
-30	-81	-18
-36	-60	-21
-3	15	51
-12	15	36
3	6	66
-27	-63	39
-30	-60	51
-30	-54	36
-42	-45	42
-39	-39	33
51	9	33
33	24	9
48	12	3
9	-84	-24
33	-78	-27
21	-84	-24

// Wang, 2021: Verb > Baseline
// Subjects=16
-23	-93	-7
-42	-60	-21
-46	9	25
-53	16	35
-57	-48	11
-8	8	53
-27	-56	42
-5	-71	28
26	-90	-7
44	-60	-21
41	-67	-1
52	16	32
7	-75	-28
-23	-90	-7
-46	-60	-11
-46	4	32
-46	23	25
-35	1	42
-8	4	56
-27	56	42
-53	-48	11
-8	-75	-28
29	-90	-7
52	16	32
7	-75	-28
-5	-56	-18
-27	-90	-11
-53	12	35
-42	19	18
-27	-56	42
-5	8	53
29	-90	-7
52	16	35
33	-48	-28

// Zhang, 2024: Verb > Baseline
// Subjects=20
12	-73	-25
12	-19	14
-36	5	44

// Wang, 2026: Verb > Baseline
// Subjects=25
-55	0	-42
-48	21	18
-38	25	14

-38	-53	-11
-6	11	56
54	-11	53
47	-21	60
36	-81	-11
29	-84	-4
-48	25	21
-45	7	28
-52	4	46
-3	14	53
-10	7	70
-27	-63	42
-48	-60	-14
-6	-70	7
5	25	39
40	-74	-14
29	-84	-4
54	35	25
36	28	11
29	-60	39

// Wang, 2026: Verb > Baseline
// Subjects=25

-48	28	5
-41	18	21
-45	11	28
-27	-88	-7
-41	-49	-18
-41	-46	-7
36	-88	-7
43	-70	-14
40	-18	63
29	-21	70
-27	-88	-7
-41	-49	-18
-48	28	14
-6	4	70
-3	14	56
-3	-63	-14
36	-88	-7
43	-60	-18
50	-14	56
36	-35	67
57	32	21
57	21	32
-34	-88	-7
-41	-49	-18
-41	-67	-14
-48	28	4
-41	18	21
-55	-11	18
47	-17	60

// Wang, 2023: Verb > Baseline
// Subjects=25

-41	-49	18
-45	-60	-14
-31	-32	-4
-52	4	46
-41	4	32
-48	14	-14
-3	-53	7
-55	-35	4
50	-14	56
29	-81	-4
36	-74	-7
33	-39	0
-41	-53	-18
-48	14	-14
-55	-4	-7
-3	7	63
-34	28	11
33	-74	-7
54	-11	53
29	-25	70
57	11	-7
20	-18	4

// Su, 2013: Verb > Baseline
// Subjects=13

45	-78	-6
33	-81	-12
-18	-93	-6
-9	72	42
9	-66	42
-45	54	51
-42	-45	57
51	-42	39
42	-54	57
-57	-30	45
54	12	9
42	3	3
0	-30	27

0	-27	45
-33	24	45
-48	0	54
-51	3	45
-57	-27	-24
-54	-39	-15
-39	-45	-24

// Luke, 2002: Verb > Baseline
// Subjects=7

-47.31	31.74	23.33
-47.51	47.72	10.55
-59.73	38.6	-17.47
-52.63	14.81	26.22
-7.94	73.33	-16.19
-1.48	27.23	-27.43
-11.05	3.15	-20.4
36.4	62.86	-15.91
60.59	30.02	10.46
-47.26	26.63	26.08
-66.02	-9.18	-11.52
47.1	15.08	-33.77
-21.35	-102.52	-1.92
33.63	-92.31	-10.59
-28.35	-52.81	-32.49
48.39	-47.87	-29.81
4.27	-83.98	-23.24
-49.4	24.6	27.44
-47.61	38.28	1.4
-52.63	14.81	26.22
0.59	42.26	52.85
-18.76	64.37	-19.6
-7.1	51.81	-39.85
64.71	46.02	-2.4
37.42	58.09	-21.06
60.87	25.51	31.07
-54.3	19.16	-19
-64.78	-28.76	-2.88
-49.32	-60.12	16.77
70.25	-13.08	-3.37
54.61	16.92	-37.45
-23.58	-101.98	-7.54
32.55	-93.38	-10.47
-3.06	-12.2	10.12
12.1	-17.36	11.49
-11.02	9.95	-16.59
-36.98	-33.08	-27.58
-30.5	-59.3	-32.94
46.28	-51.93	-27.13
13.87	-76.06	-30.91

// Li, 2004: Verb > Baseline
// Subjects=8

-52.96	36.44	5.04
-52.26	-.98	52.43
-53.48	-5.32	40.55
-40.1	17.06	-7.83
51.57	-6.38	57.92
39.79	16.93	-13.66
10.47	15.06	59.86
60.53	19.72	3.64
53.17	15.73	18.73
-42.43	-6.09	-25.67
40.59	-31.79	39.33
-50.66	-69.75	-5.79
-26.36	-63.49	36.88
-40.11	-30.02	-17.74
-17.95	-94.77	11.82
29.31	-86.8	-8.82
-18.24	-13.73	6.05
-42.29	-58.62	-24.96
29.06	-61.35	-23.66

// Chan, 2008: Verb > Baseline
// Subjects=11

-45.12	25.37	23.93
-41.86	4.76	21.43
-43.25	36.37	3.76
-18.61	17.37	-17.19
-1.5	22.05	54.89
-47.66	11.61	-8.28
-27.4	-3.02	52.21
44.41	39.95	14.24
45.44	28.7	8.61
2.69	27.61	45.3
42.6	-0.08	33.92
-39.18	-51.55	50.49
-34.81	-42.4	56.23
-24.03	-65.53	49.37
-47.5	-55.06	-9.53
4.74	29.02	37.28
-7.41	-22.31	5.59
-33.7	-81.96	-34

5.41	-78.04	-17.13

// Yang, 2011: Verb > Baseline
// Subjects=20
-28.02	-86.46	-13.48
-47.09	8.22	35.74
-49.69	39.96	7.99
-27.31	-54.91	48.38
54.58	9.4	42.86
47.58	-73.22	-13.84
2.9	12.8	59.09
29.79	-59.59	-50.74

// Yang, 2014: Verb > Baseline
// Subjects=20
-36.34	-.88	29.74
-38.9	7.05	-.14
-1.55	9.62	48.28
-55.78	-38.15	22.55
-41.22	-46.71	-23.91
-32.29	-86.06	-8.97
54.5	0.07	34.82
38.85	24.55	-.95
21.77	-87.78	-7.48
32.25	-57.29	-26.36
27.61	-61.93	-52.72

// Liang, 2006: Verb > Baseline
// Subjects=6
29.82	79.64	-19.7
24.18	68.42	44.26
34.65	-29.27	-2.28
52.23	-9.49	24.6
-36.13	73.57	-23.58
24.18	68.42	44.26
-46.92	28.06	52.83
7.19	3.79	54.31

// Yu, 2011: Verb > Noun
// Subjects=21
-51	9	6
-45	-51	9
51	-39	9
21	-63	9
24	-69	-48

// Zhang, 2018: Verb > Noun
// Subjects=21
-38	-22	50
52	-6	42
-6	-8	54
-36	-24	12
-14	-20	2
62	6	-8
68	-4	18
-54	-8	4
-64	-16	8

// Feng, 2020: Verb > Noun
// Subjects=17
-50	38	24
-26	-7	-7
-16	-79	-16
18	-81	-14
-32	40	10
30	46	8
-3	23	54
-42	15	41
-40	20	35
36	-20	-30
-49	42	29
-47	-52	-17
-44	41	5
-46	-37	-17

// Wang, 2017: Verb > Noun
// Subjects=18
54	-63	3
48	-51	3
-39	-78	15
-48	12	12
-51	-63	3
36	-81	27
